# Systematic Continental Scale Monitoring by Weather Surveillance Radar Shows Fewer Insects Above Warming Landscapes in the United States

**DOI:** 10.1111/gcb.70587

**Published:** 2025-11-18

**Authors:** Elske K. Tielens, Phillip M. Stepanian, Jeffrey F. Kelly

**Affiliations:** ^1^ Swiss Federal Research Institute WSL Birmensdorf Switzerland; ^2^ Department of Biology University of Oklahoma Norman Oklahoma USA; ^3^ Lincoln Laboratory Massachusetts Institute of Technology Lexington Massachusetts USA

**Keywords:** aeroecology, climate warming, insect monitoring, radar entomology, remote sensing, urbanization

## Abstract

Anthropogenic change is predicted to result in widespread declines in insect abundance, but assessing long‐term trends is challenging due to the scarcity of systematically collected time series measurements across large spatial scales. We develop a novel continental‐scale dataset using a nationwide network of radars in the United States to generate a 10‐year time series of daily aerial insect density and assess temporal trends. We do not find evidence of a continental‐scale net decline in insect density over the 10‐year period included in this study; instead we find a mosaic of increasing and declining trends at the landscape scale. This spatial variation in density trends is associated with climatic drivers, where areas with warmer winters experience greater declines in insect density and areas with cooling winter trends see increases in density. Winter warming has a stronger negative effect on density at higher latitudes. After assessing temporal trends, we also use the 10‐year dataset and atmospheric variables to model insect aerial abundance, finding that on a typical summer day approximately a hundred trillion (10^14^) flying insects are present in the airspace, representing millions of tons of aerial biomass. Our results provide the first continental‐scale quantification of insect density and its response to anthropogenic warming and demonstrate the utility of weather surveillance radar to provide large‐scale monitoring of insect abundance.

## Graphical abstract

Systematic remote sensed monitoring of insect density in the air shows fewer insects above warming landscapes in the United States. We estimate insect density at continental scales for the first time by leveraging the nationwide network of weather radars. On a typical summer day an estimated hundred trillion insects are in flight above the contiguous United States. At the continental scale this number has been stable for the past 10 years, while local densities increase or decline in relation to temperature trends. This study demonstrates the potential of weather radar networks for standardized large‐scale insect monitoring under global change.

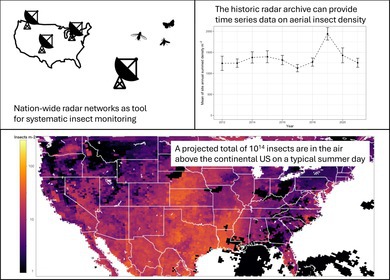

## 1 Introduction

Declines in the diversity and abundance of insects have been reported for a wide range of taxa and localities, but assessing the magnitude of this issue remains challenging due to a lack of tools for monitoring and quantifying insect abundance at scale (Didham et al. 2020; Montgomery et al. 2020). While local population declines are unequivocal and have been reported since the mid 20th century (Conrad et al. 2006; Taylor 1974), the significance of local population changes for inferring the state of global insect abundances is unclear. Evaluating large‐scale trends in insect populations is limited by the paucity of insect time series, and by the geographic and taxonomic bias in existing datasets. Specifically, most insect population surveys focus only on a subset of charismatic taxa (Brooks et al. 2012; Swengel and Swengel 2015; Wepprich et al. 2019), are conducted at a limited local scale, or have been collected in natural preserves or select ecological research stations (Crossley et al. 2020; Hallmann et al. 2017), thereby limiting our ability to identify patterns that extend across regions and species (Cardoso and Leather 2019; Lebuhn et al. 2013). Inferring broad patterns from meta‐analyses of local scale studies is complicated by differences in study design, variation in sampling effort, and disparate methods (Forister et al. 2023; Welti et al. 2021). In contrast, directly observing broad scale patterns in insect abundance and biomass requires long‐term standardized sampling across a diversity of landscapes and taxa.

Weather surveillance radar networks have the potential to provide standardized data on insect abundance at an unprecedented scale. Contrary to traditional insect survey methods, the application of remote sensing technology can provide low‐cost, low‐effort methods for long‐term automated insect surveillance that can be deployed over large spatial extents (Høye, Ärje, et al. 2021). In particular, radar technology has a longstanding history of supporting entomological applications by measuring large‐scale movements of flying insects (Hu et al. 2016; Stepanian et al. 2020; Tielens et al. 2021). The use of radar to quantify animal movement has been extensively discussed and validated elsewhere (Chapman et al. 2002; Lukach et al. 2022; Stepanian et al. 2016), and is widely used in ornithology (Nilsson et al. 2019; Van Doren and Horton 2018). Specifically, weather radar has allowed ornithologists to quantify abundances of birds in flight, declines in abundance, and their potential drivers (Deng et al. 2023; Dokter et al. 2019; Lin et al. 2019; Rosenberg et al. 2019; Van Doren et al. 2017).

For many insect taxa flight is an important life history strategy, and insects use aerial habitats at different altitudes for various behaviors. While daily foraging activity or local dispersal takes place at lower altitudes within the atmosphere (within several meters of the ground), that is, within an insect's so‐called “flight boundary layer” (Srygley and Dudley 2007), some taxa engage in high‐altitude flight visible on weather surveillance radar. High‐altitude flight includes dispersal, mating flights, or seasonal migration, and although only a subset of taxa engages in such flights, these represent most insect orders and a diversity of life histories (Chapman et al. 2004; Glick 1939). Moreover, the mechanism of aerial dispersal connects communities from local to regional scales and provides a flux of energy, biomass, and nutrients across landscapes (Stepanian et al. 2020). This movement impacts human and natural systems through species interactions and ecosystem functioning (Hu et al. 2016; Satterfield et al. 2020); for example, migratory hoverfly species in the UK provide important pollination services and perform pest control by consuming 3–10 trillion aphids annually (Wotton et al. 2019). As such, surveying insects in flight using radar is both methodologically efficient and monitors activity during a consequential life stage. The use of weather radar allows quantification of insect density across the contiguous United States, providing insight into spatial and temporal patterns of insect density, and the role of potential stressors such as land use and climate change.

Anthropogenic warming is a key stressor, and differences in its effects on species' survival, growth rates, phenology, or species interactions produce complex and non‐additive biodiversity and abundance patterns. Warming may increase insect population sizes through expanded accumulated degree days, shortened generation time, and range expansion. For example, pest population growth and outbreak frequency are projected to increase under warming conditions, threatening food security and resulting in agricultural losses (Deutsch et al. 2018). In contrast, warming conditions can also produce physiological stress, reduce survival, and result in phenological mismatches (Visser and Holleman 2001), or habitat loss for thermally restricted species (Fox et al. 2014). Additionally, responses to changes in temperature or precipitation patterns are species‐specific (Forister et al. 2021). Thus while direct impacts of warming have been well studied, how these local effects interact to produce large‐scale temporal patterns in insect abundance or potential declines across taxa has not been documented (Didham et al. 2020; Wagner 2020; Wepprich et al. 2019).

In this study we document spatial and temporal trends in insect density at an unprecedented scale by leveraging the widespread monitoring of weather surveillance radar. We use the near‐continuous coverage of the United States weather surveillance radar network to generate a novel 10‐year time series dataset of insect density aloft across the contiguous United States. We expand upon existing applications of radar quantifying insect abundance aloft (Hu et al. 2016; Stepanian et al. 2020; Tielens et al. 2021) with a data processing workflow that applies this methodology at a continental scale. This approach allows us to map insects in the air, project across the US, estimate declines in the density of high‐altitude insects, and explore large‐scale global change drivers.

## 2 Materials and Methods

### 2.1 Weather Radar Network

We used the historical archive of weather surveillance radar and their near‐continuous monitoring to examine spatial and temporal patterns in day‐flying insect density. We developed a workflow to generate a dataset of day‐flying insect density using the next‐generation weather surveillance radar (NEXRAD) system, a network of high‐resolution Doppler weather radars (WSR‐88D) operated by the NOAA National Weather Service (NWS), the Federal Aviation Administration and the U.S. Air Force. Use of the NEXRAD radar system for ecological applications has been widely discussed elsewhere (Diehl and Larkin 2005; Larkin 2012; Stepanian et al. 2016). The NEXRAD radar stations operate at S‐band, with a frequency of 2.7–3.0 GHz and an approximate 10.7 cm wavelength. We applied our workflow to 140 radar stations in the contiguous United States for the period for which dual‐polarized radar data has been archived (2012–2021). We omitted two radars for which data quality issues such as systematic contamination by chaff could not be excluded (Methods S1). The NWS provides technical details on the NEXRAD system (https://www.ncei.noaa.gov/products/radar/next‐generation‐weather‐radar), and radar data is archived and publicly available from Amazon Web Services (https://noaa‐nexrad‐level2.S3.amazonaws.com).

### 2.2 Data Generation

We generated data on insects aloft using weather surveillance radar following workflows outlined in previous work (Tielens et al. 2021; Tielens and Kelly 2024). In short, we extract insect density from the radar reflectivity factor for each volume scan, producing a continental‐scale spatial and temporal dataset of day‐flying insect density. To avoid double counting individuals in subsequent volume scans, we use a single daily radar scan to provide a representative minimum daily value. This method results in insect densities that are an underestimate of the true daily density, but an appropriate snapshot for comparison in our analysis of spatial and temporal variation in insect density. We constrain the timing of our data to the radar scan nearest to noon to reduce contamination from non‐insect sources. The initial radar product dataset was generated using Python 3.7.4, and processed as described in previous work (Stepanian et al. 2020). We use the package PyEphem to identify local solar noon, and the package Py‐ART (Helmus and Collis 2016) to extract the radar volume scans nearest to noon from the NWS NEXRAD archive. We subset to non‐redundant sweeps. We convert radar reflectivity factor (dBZ) to total scattering area (cm^2^) and scattering area density (cm^2^/km^3^) in each resolution volume (Chilson et al. 2012).

We constructed a ground clutter filter to remove interference from ground structures in proximity to the radar. We developed this clutter filter by downloading at least 101 scans for every radar and year during the first 15 days of January, when biological scatter should be minimal in the northern hemisphere. From these scans we plotted the cumulative reflectivity for every pixel, set the 85th percentile of the distribution of reflectivity values as a threshold, and considered pixels where the cumulative value was more than this 85th percentile threshold a “permanent” feature. These pixels were marked and included in a mask, which is then applied to all scans. Pixels with “permanent” features were removed from the scan.

Next, we identified and removed non‐arthropod signals on the radar scans based on several radar products. Differential reflectivity is the ratio of horizontal to vertical polarized equivalent reflectivity factor, providing information on the aspect ratio. Correlation coefficient is given by the cross correlation between the time of arrival of horizontal and vertical polarized waves. Together, these variables allow reasonable distinguishing between round water drops in the air and biological signals of varying shapes (Kilambi et al. 2018). We used differential reflectivity and correlation coefficient to calculate depolarization ratio. We excluded weather signals by removing pixels with depolarization ratio < −12.5. Reflectivity and differential reflectivity are key parameters in differentiating insect signals from avian and non‐biological signals (Gauthreaux and Diehl 2020). Insect signals on weather surveillance radar are characterized by low reflectivity and high differential reflectivity (Stepanian et al. 2016). We excluded pixels with reflectivity > 40 dBZ and with differential reflectivity < 5 dB. This process resulted in a scatter density of insects for each pixel in the volume scan.

We quantify insect numbers in terms of scattering density to be able to use this data for spatially explicit models and to best deal with variation in the number of pixels included after filtering. The radar beam geometry under standard refraction conditions provides a minimum beam‐center height of approximately 19 m above ground level. In regions of the US with minimal topography and structures, reliable insect surveillance is achieved at all altitudes above this threshold. We generate an altitudinal profile of scattering density by taking the mean density in 50‐m altitude bins up to 3 km above ground level (excluding weather or other non‐sampleable pixels) and integrate over all altitudes to obtain column‐summed scattering density (cm^2^/km^2^). We convert scattering density (cm^2^/km^2^) to insect density (insects/km^2^) assuming the most common day‐flying insect scatterers are small insects (Drake and Reynolds 2012; Riley 1985). Combining information from the literature on aerial sampling of insects in the flight boundary layer and radar cross section measurements on S‐band radar, we assume mean RCS at 1 × 10^−5^ cm^2^ (Contreras and Frasier 2008; Drake and Reynolds 2012). This RCS corresponds to small insects commonly aloft during the day including Aphididae (Hemiptera), pollen beetles (e.g., *Meligethes aeneus* , Coleoptera: Nitidulidae), hoverflies (e.g., *Eristalis tenax* , Diptera: Syrphidae), braconid wasps (e.g., *Aphidius nigripes*, Hymenoptera: Braconidae), small flies such as Agromyzidae (e.g., *Agromyza virens*, Diptera: Agromyzidae), and thrips (e.g., *Frankliniella* sp., Thysanoptera: Thripidae) (Chapman et al. 2004; Glick 1939).

Our filtering was efficient in removing the main expected sources of non‐insect signal (precipitation), and we conducted quality control by calculating weekly mean biomass and removing all data points greater than two standard deviations from the weekly mean. After data cleaning, we manually checked remaining outliers and peak events for quality control, removing data points for days showing non‐typical insect images (see Supporting Information: Appendix Methods for more details). While these data cleaning steps did not affect the overall temporal and spatial patterns in our data, we retained the most conservative filtering.

Radar stations across the United States were dual‐polarized between 2011 and 2013. For each individual station we included only dual‐polarized data, to allow for filtering of non‐arthropod biological scatter. To ensure complete seasonal patterns, we removed any year for a site from the dataset at sites where dual‐polarization was not completed prior to May 1 for that year. This resulted in varying lengths of observation time series (*n* = 51 for 10 years, *n* = 79 for 9 years, *n* = 10 for 7 years). We evaluated how well the data met spatial and seasonal expectations based on general ecological patterns for large scale distributions of insect abundance (see Supporting Information: Appendix Methods, Quality control).

### 2.3 Projecting From North American Regional Reanalysis

To model insect density in the air, we followed standard and previously validated methods for extending animal abundance estimates into areas with insufficient radar coverage, applying gradient‐boosted trees to predict insect density on radar from atmospheric conditions (Van Doren and Horton 2018). We used atmospheric conditions reported by the North American Regional Reanalysis (NARR) (Mesinger et al. 2006), which compiles various data sources to produce an estimate of weather conditions in 3‐h intervals in a 32 km pixel size grid across North America. We extracted daily NARR data for the time point closest to noon and the NARR grid cell closest to the radar site. We utilized 77 variables from the NARR data set for the period 2012–2021. We used gradient‐boosted trees to relate atmospheric conditions to observed insect density on radar following methods described in detail in Van Doren and Horton (2018). In summary, we used the gradient boosting framework XGBoost (Chen and Guestrin 2016) in Python. We trained the model on NARR weather data with column‐summed insect density as the response variable, randomly splitting the data into 75% training and 25% testing. We set model parameters to the following values, which were shown to function as the best performing combination in prior work that linked weather radar observations to environmental predictors (Van Doren and Horton 2018): learning rate (eta) = 0.01, maximum tree depth = 12, gamma = 1, min_child_weight = 5, colsample_bytree = 1 and subsample = 0.7, and ran this for 1000 rounds. The variance described by NARR predictor variables in the full model is given in the supplement (Figure S2).

### 2.4 Data Analysis

#### 2.4.1 Insect Response Variables

We took two approaches to analyze spatial patterns in day‐flying insect density across the United States. After calculating annual site means for each year, we quantified insect density anomaly for each site and year combination, and we quantified the trend in insect density for each site. Insect density anomaly allows us to analyze the role of climate in driving year‐to‐year variation in density at a given site. The second approach is to quantify the change in insect density over time, which allows identification of regions where insects are experiencing declines and analyzes climate or land use correlates with this trend.

To quantify both of these measures we calculated the mean, standard deviation and standard error of day‐flying insect density for each radar site for the period 2012–2021. For each site, we first quantified insect density anomaly by calculating overall mean density across the 10‐year period and then yearly deviation from site mean density. To quantify site‐specific temporal trends in insect density over the 10‐year study period, we generated a linear model of density as a function of year for every radar station. To account for differences between sites in overall insect density when assessing temporal trends we used the linear model coefficients to calculate percentage change relative to mean annual site density.

#### 2.4.2 Climatic and Land Cover Drivers

We extracted monthly data on local climatic conditions from WorldClim for each radar station for the period 2012–2021 (Fick and Hijmans 2017). To calculate the 10‐year change in climate (mean spring, summer, fall, and winter temperature) we used the slope of site‐specific linear regressions, as well as the slope for the 10‐year trend in annual precipitation. We calculated anomalies in seasonal temperature and precipitation relative to 30‐year averages. To generate land cover predictors, we used the 2016 National Land Cover Database, which provides nationwide data on categorical land cover at a 30 m resolution (Dewitz 2019). We generated land cover data for each site in R using packages raster, sf, and exactextractr (Baston 2022; Hijmans 2022; Pebesma 2018). We extracted the fraction of the landscape occupied by each land cover type within 80 km of each radar. We explored other scales for the landscape analysis (40 km radius, 100 km radius) and found similar results; therefore we selected 80 km as best fitting the scale of our radar data.

### 2.5 Modeling

We evaluated drivers of interannual variation in insect density by analyzing annual deviation in insect density from long‐term site means as a function of climate anomaly. We used a model averaging approach (see Supporting Information); the global model was a linear regression with the following variables: 30‐year temperature anomaly for winter, spring, summer, and fall, precipitation anomaly for all four seasons, latitude, longitude, and year, with a random effect for site ID. We averaged estimates for all models within < 4 of the lowest AIC model.

We analyzed drivers of temporal trends in insect density using 10‐year percentage change as response variable. Percentage change data often exhibit positive skew; we assessed this for the trend in insect density and found that skew did not affect model interpretation (skewness = 1.17, see Supporting Information for how model assumptions were evaluated and handled). We evaluated year, latitude, and longitude individually as potential drivers of 10‐year trends in insect density with linear regression models. We evaluated the role of climate predictor variables using a model averaging approach, averaging estimates for all models within < 4 of the lowest AIC model. The global climate model included trend in mean spring temperature, mean summer temperature, mean fall temperature, and mean temperature during the preceding winter, as well as mean trend in annual precipitation. We explored interaction effects between climate trends and latitude or longitude and found a significant interaction for latitude with winter warming. We evaluated the role of land cover predictor variables using a model averaging approach, averaging estimates for all models within < 4 of the lowest AIC model, with the global model including fraction land cover for the most common land cover types; forest, grassland, shrub, aquatic, developed, crop, and pasture (Figure S6).

Land cover types vary in their winter warming trends, and we used partial correlation coefficients to assess the effect of land cover drivers while controlling for winter warming trends. We included partial correlation for all land cover types significantly associated with insect density (Table S4). We calculated partial correlation coefficients to assess the effect of land cover on insect density trends while controlling for winter warming, and to assess the effect of winter warming on insect density trends while controlling for land cover (package ppcor in R) (Kim 2015).

Lastly, we separately explored the role of other potential landscape associations with variation in insect density trends, including biome, net primary productivity, population density, human footprint index, and discuss these in detail in the supplement (Methods S1).

## 3 Results

### 3.1 Systematic Monitoring of Insect Density

**FIGURE 1:**
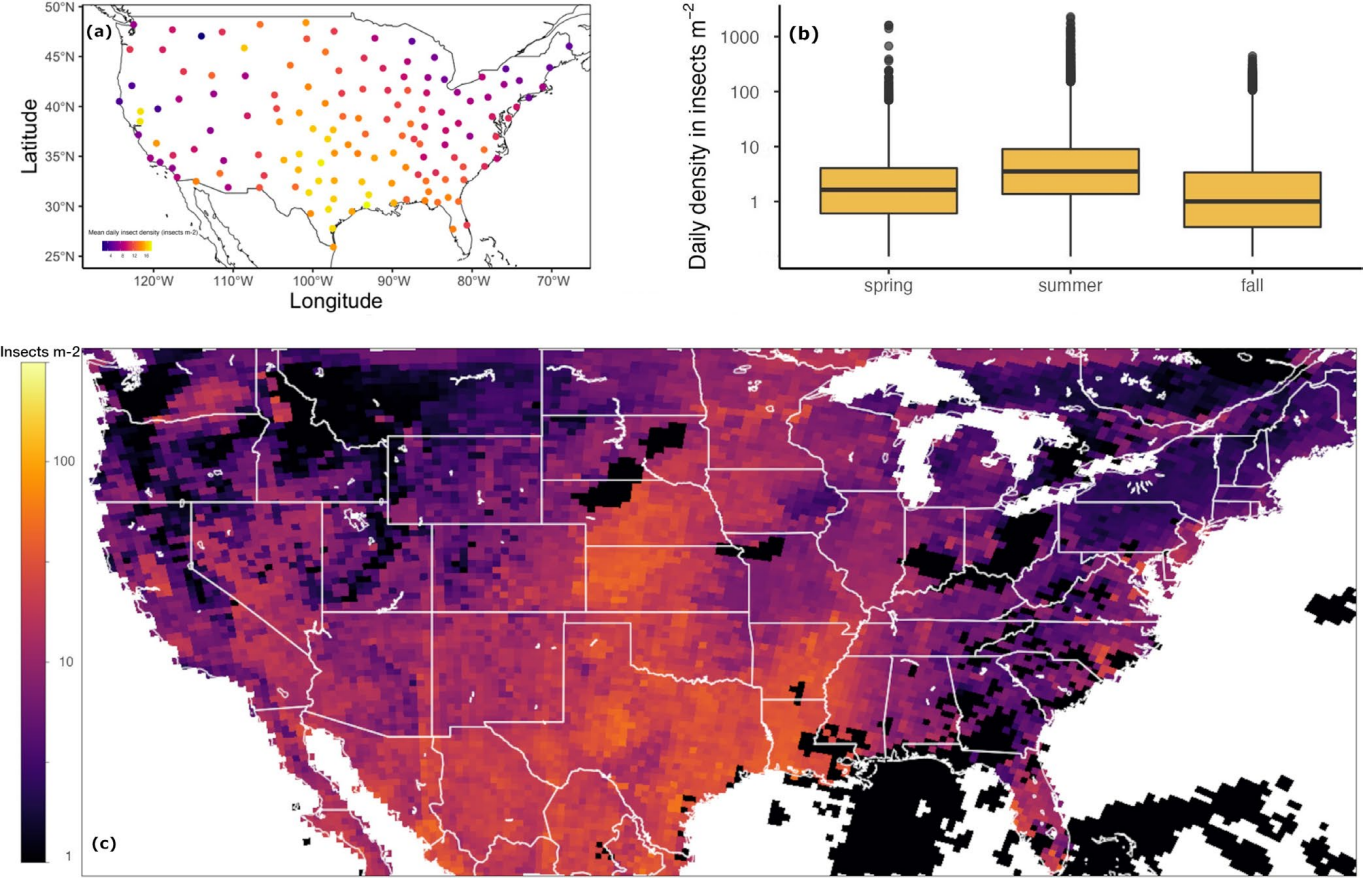
Day‐flying insect density above the contiguous United States. (a) Observed mean insect density per radar snapshot in insects m^−2^ at NEXRAD weather surveillance radar sites. (b) Boxplots of the distribution and seasonal mean of insect density per snapshot across all sites for spring, summer, and fall, in insects m^−2^. (c) Projected day‐flying insect density for a given summer day (August 25, 2021), modeled from observed insect density and North American Regional Reanalysis atmospheric variables (Mesinger et al. 2006). Color represents the vertically integrated number of insects m^−2^, with lighter colors indicating higher density. Areas with precipitation on 25 August 2021 are marked in black. Map lines delineate study areas and do not necessarily depict accepted national boundaries.

Mean noon‐time observed density was 4.3 insects m^−2^ (summed for the 3 km high column of air above each square meter), varied across the contiguous United States, and was greatest in the Gulf Coast region in the southern United States (Figure 1a). Observed day‐flying insect density declined with latitude (Figure S1a, latitude est. = −3.1, *p* < 0.0001), and varied unimodally with longitude (Figure S1b). Flying insect density varied seasonally and was highest during summer (Figure 1b). We combined the 10‐year archive of daily radar snapshot observations of insect density with atmospheric variables to project abundance across the contiguous United States, and atmospheric conditions explained 40.5% of the variance in insect density. A projected total of 1.0 × 10^14^ day‐flying insects were present in the air above the contiguous United States on a typical summer day (Figure 1c; estimated for August 25, 2021). To avoid compounding uncertainty, we continue further analyses here on observed column‐summed insect density (insects m^−2^) within the site (radar) domain rather than on projected insect abundance.

### 3.2 No Net Continental Scale Decline

**FIGURE 2:**
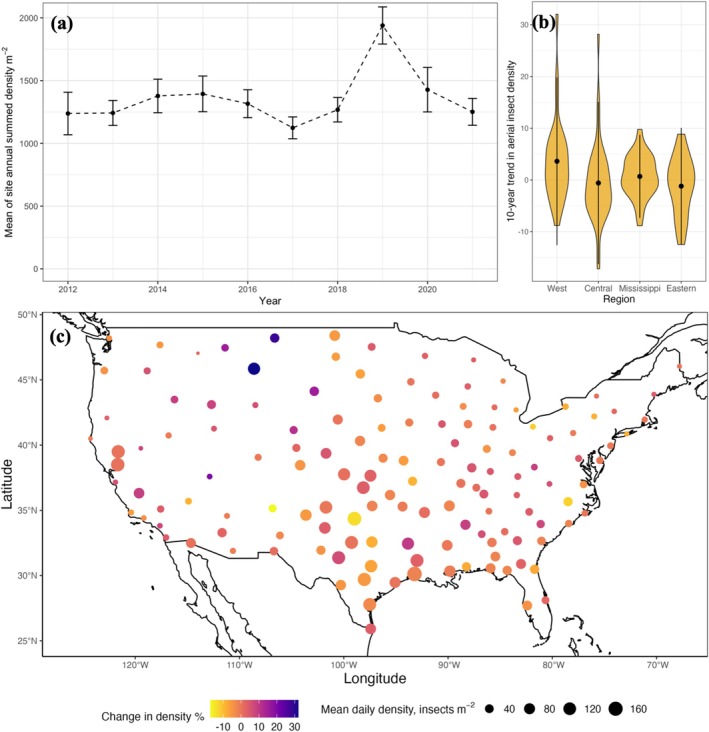
Patterns in the change in insect density over the period 2012–2021. (a) Annual day‐flying insect density over time in insects m^−2^, mean annual density (black dots), and standard deviation (black lines). (b) Violin plot of the distribution of temporal trends in site day‐flying insect density across geographic region, with points indicating regional mean. (c) Map of change in insect density over the 10‐year period across the contiguous United States. Point color indicates percentage change in insect density, size indicates mean site daily insect density in insects m^−2^. Map lines delineate study areas and do not necessarily depict accepted national boundaries.

At continental scale, the trend in day‐flying insect density was stable over the period 2012–2021, with high interannual variation (Figure 2a, est. = 0.45, *p* > 0.05). Locally, temporal trends varied spatially even among geographically adjacent sites (Figure 2b,c), with 52% of sites experiencing increases in density and 48% of sites showing decreases in density (Table S5). Increasing trends over time were most common in the northern Plains and Western United States while declines in day‐flying insect density predominantly occurred in coastal areas. Change in insect density over time did not vary systematically with latitude or longitude (Figure S5, latitude est. = 0.23, *p* = 0.077; longitude est. = −0.071, *p* = 0.14).

### 3.3 Warming Trend

We evaluated interannual variation in insect density per site by comparing mean annual insect density to the 10‐year average density at each radar station. Interannual variation, that is, the distribution of density anomaly around site long‐term mean, differed strongly across sites (Figure S3). Year was not a significant predictor of interannual variation in insect density; instead, this variation was associated with climate anomalies (Table 1). Higher‐than‐average insect density occurred in years and sites with mean summer temperatures warmer than the 30‐year mean (est. = 1437, *p* < 0.01). Deviation from mean site insect density was negatively associated with winter temperature anomaly during the preceding year (est. = −426.3, *p* < 0.01). Lastly, day‐flying insect density was higher in years with wetter‐than‐average spring conditions (est. = 11.5, *p* < 0.01). Model averaging also retained latitude, longitude, year, 30‐year temperature anomaly for mean spring and mean fall temperature, and winter, summer, and fall precipitation anomaly, but these were not significant predictors (model averaging for the global model containing all climatic variables; all were retained at ΔAIC < 4).

**TABLE 1:** Model averaging output for modeling interannual variation in insect density (annual deviation from 10‐year mean) as a function of climate anomaly conditions, showing predictor variables weighted average coefficients, appearance frequency, and importance across all candidate models within ΔAIC<4. *p*‐values are given * (<0.05), ** (<0.01), *** (<0.001).

Climatic predictor	Weighted average coefficient	Appearance frequency	Sum of weights
Latitude	−4.75	0.396	0.37
Longitude	−0.567	0.281	0.2
Year	7.60	0.385	0.32
Fall temperature anomaly	−16.5	0.302	0.26
Spring temperature anomaly	−33.6	0.438	0.43
Winter temperature anomaly	−39.0**	1	1
Summer temperature anomaly	136***	1	1
Fall precipitation anomaly	−0.296	0.667	0.7
Spring precipitation anomaly	0.729***	1	1
Winter precipitation anomaly	0.0445	0.271	0.2
Summer precipitation anomaly	−0.250	0.708	0.75

**FIGURE 3:**
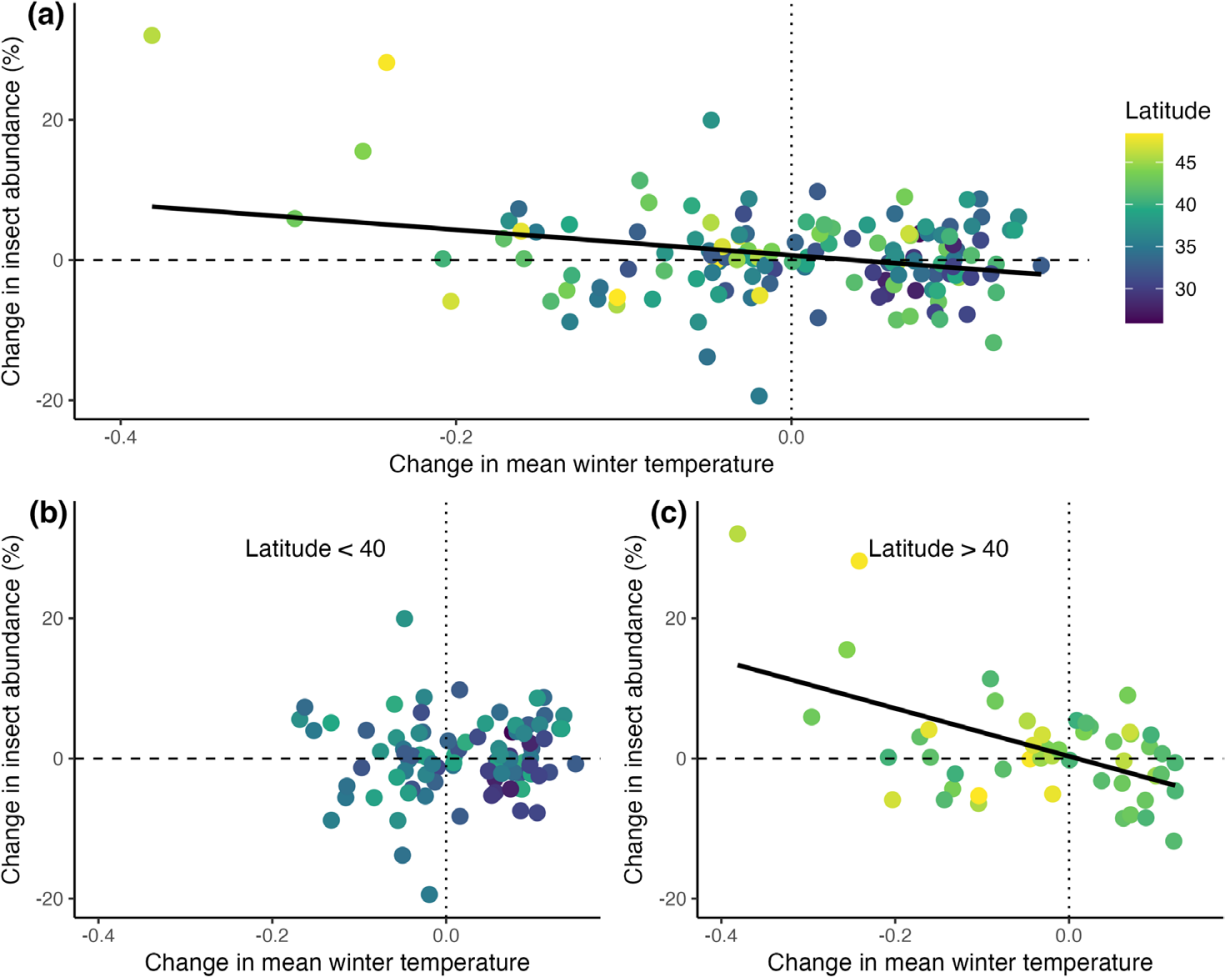
Temporal pattern of change in insect density as a function of change in winter temperature. (a) 10‐year trend in day‐flying insect density as a function of the change in local mean winter temperature, colored by site latitude. (b) Temporal trend as a function of winter temperature at latitudes ≤ 40°. (c) Temporal trend as a function of winter temperature at latitudes > 40°. Fitted lines are derived from a least‐squares linear regression on percentage change in insect density. Linear model with change in mean winter temperature, interaction with latitude, and longitude explains 18% of variation in insect declines.

Besides year‐to‐year variation in insect density, temporal trends in insect density were also associated with winter warming trends (Figure 3a). To evaluate spatial patterns in the effects of climate on change in insect density over time we quantified the slope in insect density over the 10‐year observation period (i.e., Figure 2c) and analyzed these against the trend over the same time period in mean temperature for each season and the trend in annual precipitation. The trend in insect density was negatively associated with the trend in mean winter temperature, with declining insect density at sites with warming winter conditions (Figure 3a, est. = −21, *p* < 0.001). The effects of warming winter temperature on insect density trends varied with latitude. High latitude sites showed a strong decline in insect density over time with warming winter conditions (Figure 3c), while at low latitudes the trend in insect abundance was not associated with winter temperature trends (Figure 3b). We analyzed drivers of the trend in insect density using AIC and all models within ΔAIC < 4 included the trend in mean winter temperature, latitude, and the interaction between them. No other climatic trends were significantly associated with the trend in insect density, although latitude, longitude, the trend in spring, summer, and fall temperature, and the trend in annual precipitation were retained from the global model in model averaging (Table 2; ΔAIC < 4).

**TABLE 2:** Model averaging output for modeling 10‐year trend in insect density as a function of temporal trend in climatic conditions, showing predictor variables weighted average coefficients, appearance frequency, and importance across all candidate models within ΔAIC<4. *p*‐values are given * (<0.05), ** (<0.01), *** (<0.001).

Climatic predictor	Weighted average coefficient	Appearance frequency	Sum of weights
Latitude	0.0360	0.4	0.33
Longitude	0.00161	0.2	0.14
Trend in mean fall temperature	0.384	0.2	0.14
Trend in mean spring temperature	−0.820	0.267	0.18
Trend in mean winter temperature	−21.0***	1	1
Trend in mean summer temperature	0.818	0.267	0.18
Trend in mean precipitation	0.190	0.133	0.11

### 3.4 Land Cover Analysis and Urban Warming

**FIGURE 4:**
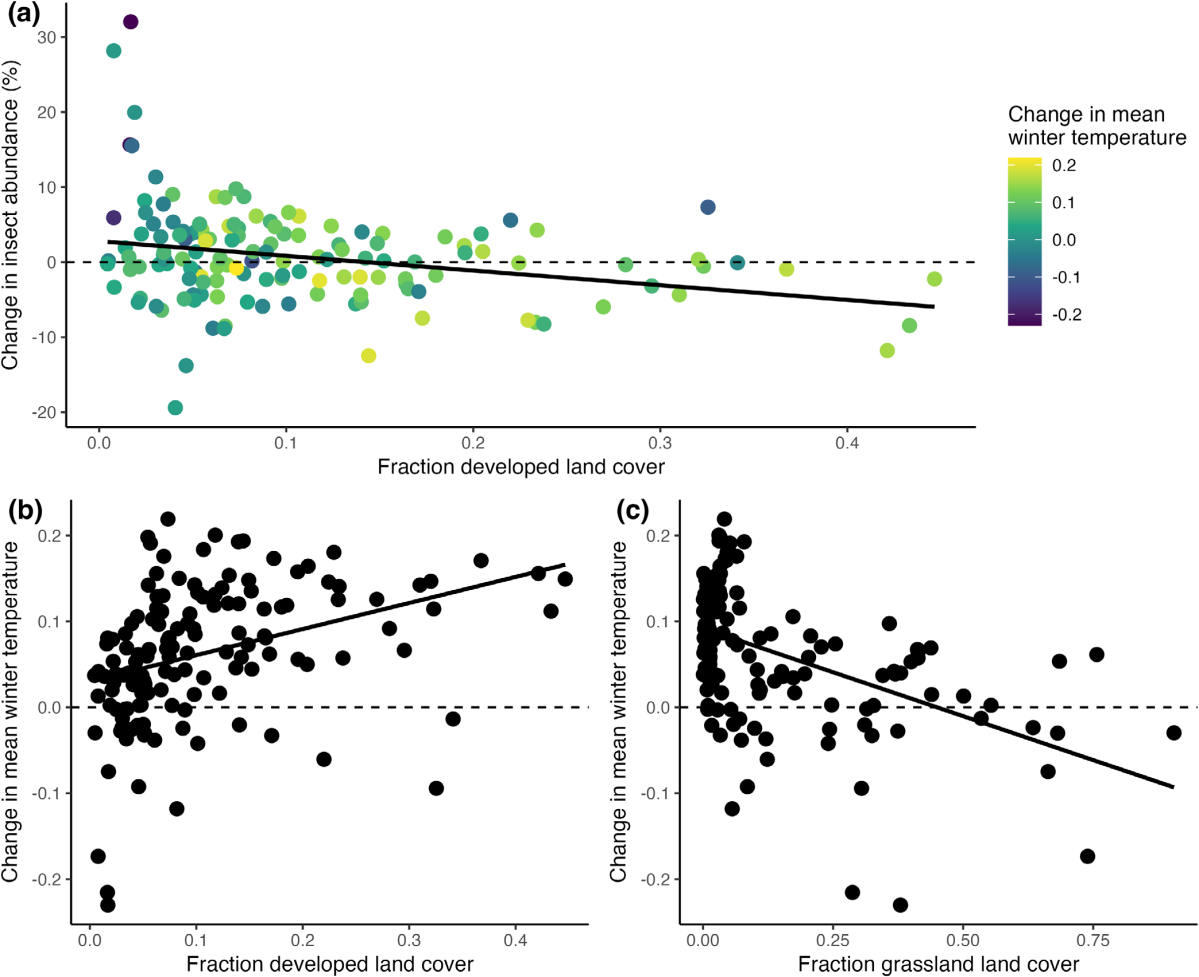
Temporal pattern of change in insect density as a function of developed land cover. (a) 10‐year trend in day‐flying insect density as a function of the fraction developed land cover in the landscape, colored by the change in mean winter temperature. Line is given by LM. (b) Change in mean winter temperature as a function of the fraction developed land cover. Line is given by LM, correlation coefficient = 0.37 *p* < 0.0001. (c) Change in mean winter temperature as a function of the fraction grassland in the landscape. Line is given by LM, correlation coefficient = −0.54, *p* < 0.0001.

Change in day‐flying insect density was negatively correlated with developed land cover, such that areas with a greater fraction of developed landscape experienced greater declines in insect density over time (Figure 4a, est. = −16.1, *p* < 0.05). We conducted model averaging with fraction land cover for all NLCD variables, including developed, pasture, cropland, aquatic, forested, shrubland, and grassland land cover. The average model (ΔAIC < 4) retained all land cover variables but found a significant relationship only with developed land cover (Table 3, Figure S6). Besides land cover type, insect density was also negatively correlated with the human footprint index (Venter et al. 2018) (Figure S7A, est. = −0.29, *p* < 0.001), but was not correlated with human population density alone (Figure S7B), suggesting complex forces resulting from multiple anthropogenic pressures drive spatial variation in insect declines (Seibold et al. 2019).

**TABLE 3:** Model averaging output for modeling 10‐year trend in insect density as a function of land cover variables, showing predictor variables weighted average coefficients, appearance frequency, and importance across all candidate models within ΔAIC<4. *p*‐values are given * (<0.05), ** (<0.01), *** (<0.001).

Land cover predictor	Weighted average coefficient	Appearance frequency	Sum of weights
Developed land cover	5.24	0.810	0.88
Grassland cover	7.18	0.643	0.7
Forest cover	−16.2*	0.476	0.48
Pasture cover	−10.5	0.524	0.49
Aquatic land cover	−0.95	0.381	0.29
Cropland cover	−3.22	0.357	0.26
Shrubland cover	−1.75	0.333	0.25

Winter warming trends varied across land cover types (Table S3). Developed landscapes experienced disproportionate winter warming trends in the study period (Figure 4b, corr. coeff. = 0.35, *p* < 0.0001), while landscapes with a greater grassland fraction more commonly showed cooling trends (Figure 4c, corr. coeff. = −0.49, *p* < 0.0001). Both winter temperature trends and urbanization were independently associated with insect density trends. After correcting for developed land cover, winter warming trends remained significantly correlated with the change in insect density (Table S4, Pearson partial corr. coeff. = −0.27, *p* < 0.01). Similarly, developed land cover was significantly correlated with insect trends after correcting for winter temperature trend (Pearson partial corr. coeff. = −0.19, *p* < 0.05). In contrast, after correcting for the partial correlation with winter warming, fraction grassland land cover was not significantly correlated with insect density trends (Pearson partial corr. coeff. = 0.1). The correlation between fraction land cover and winter warming trend suggests that urbanization‐induced warming plays a role in the change in insect density.

## 4 Discussion

### 4.1 What Drives Spatial Patterns in Insect Density Trends?

This study forms the first continental‐scale time series analysis of insect density in the United States. We do not find evidence for a large‐scale decline in day‐flying insect density over the 10‐year period included in this study, in contrast with recent studies (Hallmann et al. 2017; van Klink et al. 2020). Instead, we find a net stable trend in insect density at the continental scale, which is formed by a spatial mosaic of increasing and declining trends at the local scale. The literature unambiguously shows that local population declines are widespread and occur across diverse taxa (Forister et al. 2021; Wepprich et al. 2019), continuing to be of conservation concern. However, these local trends do not appear to scale up to continental declines in flying insects. This mismatch may be because regions experiencing local increases in insect abundance are buffering continental trends. Variable responses at large scales can also result from a lack of spatial synchrony in time series trends, which has been demonstrated in several regional studies of insect abundance (Bell et al. 2020; Crossley et al. 2020; Lewinsohn et al. 2022).

Trends in day‐flying insect density over the past decade vary spatially, with some areas experiencing increases and some areas experiencing declines. This pattern of disparate abundance trends between sites in close proximity to each other is consistent with a previous meta‐analysis on temporal abundance trends in North America (van Klink et al. 2020), and underscores the importance of standardized monitoring to assess large‐scale patterns. While comprehensive efforts to assess population trends in the US exist for specific taxa (Forister et al. 2021) (i.e., the North American Butterfly Association counts), no such efforts exist for a broad taxonomic range of insect species. Weather surveillance radar data fills this gap for continental‐scale insect monitoring as well as providing myriad opportunities for future analysis of regional or local patterns in insect abundance, temporal trends, and their drivers.

### 4.2 Anthropogenic Stressors

Although we do not observe continental scale declines, the spatial patterns of abundance trends identified in this study can pinpoint potential stressors or drivers of insect declines. Declines in aerial insect density were stronger in regions that experienced increasing winter temperatures. During overwintering, warming can decrease fitness by releasing organisms from cold‐induced dormancy, thereby increasing metabolic rates, and depleting energy reserves (Pelini et al. 2009; Williams et al. 2012). Winter warming may also result in increased mortality due to phenological mismatches with resources (Visser and Holleman 2001), and may extend the activity period for natural enemies and reduce pathogen die‐off during the winter season (Pekár et al. 2015). Negative effects of winter warming on insect abundance in temperate regions have been shown in local surveys of beetles (Harris et al. 2019), butterflies (Conrad et al. 2006; Fox et al. 2014; Sparks et al. 2007), and arthropods generally (Fitzgerald et al. 2021; Seibold et al. 2019), indicating that winter is a particularly sensitive season for temperate ectotherms.

Sensitivity to winter warming varies across populations and is likely more common in cooler climates where thermal seasonality is strong (Pelini et al. 2009; Post et al. 2018; Williams et al. 2003). Our results show a negative effect of winter warming at high latitudes, with no effect at latitudes below 40°. This latitudinal interaction between winter warming and aerial insect density aligns with theory suggesting that climate warming will have the strongest effect on cool‐adapted arthropods (Fitzgerald et al. 2021). For example, metabolic costs are greater at high latitudes (Kukal et al. 1991), affecting organisms' cold tolerance and resulting in greater risks of energy depletion if winters become warmer under global change. Experimental warming has shown that high elevation gall wasp species experience greater decreases in survival and fecundity than those from lower latitudes (Williams et al. 2003). These stronger responses from high latitude insects to winter warming are particularly concerning because the magnitude of warming under climate change also increases with latitude (Masson‐Delmotte et al. 2021; Post et al. 2018).

Beyond long‐term trends, we observed strong variation in insect density from year to year in response to climatic variables. Interannual variation was correlated with local climate anomalies, congruent with a recent smaller‐scale study of flying insect abundance (Müller et al. 2024). We found that insect density was higher than average at locations where summers were warmer or where springs were wetter than the local long‐term average, while local insect density was lower after warmer‐than‐average winters. Müller et al. reanalyzed 27 years of data on flying insects captured in malaise traps in Germany, finding that insect biomass was positively correlated with warmer‐than‐average growing season temperatures and wetter‐than‐average springs, but negatively correlated with winter temperature anomaly (Müller et al. 2024). While this study and our study were conducted on different continents and with sampling methods operating at vastly different scales, the similarities suggest that insect movement at ground level and at high altitude may be governed by similar drivers.

Declines in aerial insect density were associated with greater anthropogenic development as well as warming conditions. This finding is consistent with a global meta‐analysis finding a negative relationship between long‐term trends and landscape‐scale urbanization (van Klink et al. 2020), and a study of population trends across habitats in the UK (Bell et al. 2020). Locally, increased anthropogenic development can cause insect population declines through various mechanisms (Fenoglio et al. 2021), including habitat loss and changes in landscape configuration (Corcos et al. 2019; Piano et al. 2020), and light pollution (Wilson et al. 2018). Urban heat islands also result in increased temperatures, with documented negative effects on insect populations (Hamblin et al. 2018). Our analysis showed a correlation between winter warming and developed land cover, suggesting urban heat islands may play a role in spatial patterns of insect declines. While regional differences in historic land use patterns and the relative density of built environments likely result in regionally dissimilar insect abundance trends, increasing anthropogenic development is a major concern for insect populations broadly.

### 4.3 Considering Taxonomic Diversity

The discrepancy between local population‐level declines and this study's finding of stable insect abundance at the continental scale may result from summing across taxonomic “winners” and “losers”; where increases in the abundance of certain species, such as common taxa or pests, compensate for declines in others (though see Van Klink et al. 2024). Pests and common species are frequently not the focus of long‐term surveys, which may result in skewed assessments of general insect abundance if the response of such species to anthropogenic change differs. Varying time series trends across species are common, with several multi‐species occupancy studies demonstrating patterns of both increases and declines over time (Lamarre et al. 2022; Macgregor et al. 2019; Wagner et al. 2021). For example, bee occurrence records from the past 140 years in the US show declines in 29% and increases in 27% of species (Bartomeus et al. 2013). Moreover, responses to global change stressors are expected to be species‐specific; for example, previous studies have found that the urban heat island effect decreases abundances of native bees (Hamblin et al. 2018) while increasing densities of urban tree pests (Dale and Frank 2017). Responses may also depend on species' traits; for example bumblebee species with narrower climatic niches are more vulnerable to decline (Williams et al. 2007), and recent declines in butterflies have disproportionately occurred in habitat specialists (van Strien et al. 2019; van Swaay et al. 2006). Integrating species‐specific population studies with continental‐scale surveillance technologies such as radar can provide temporal and spatial resolution to understand species distributions, responses to environmental drivers, and taxonomic patterns (Nilsson et al. 2021).

High‐altitude day‐flying insects form only a limited taxonomic subset of the diversity of insects. However, they are good indicators for tracking widespread insect abundance as they include taxa from nearly every insect order (Chapman et al. 2004; Glick 1939), with disparate life histories and traits (e.g., trophic level, degree of host specificity, developing in aquatic and terrestrial habitats). This group includes herbivore pests (Hemiptera: Aphididae, Thysanoptera), fungal feeders (Coleoptera: Corticaria), predators (Diptera: Syrphini), generalist and specialist parasitoids (Hymenoptera: Ichneumonidae), and insects with other feeding strategies [see Chapman et al. (2004), Glick (1939), and Hardy and Milne (1938) for a fuller list of taxa collected using aerial sampling]. Although the size distribution of high‐altitude diurnal insects skews toward micro‐insects, there are notable large‐bodied examples (i.e., green darner dragonflies, Lepidoptera such as monarchs and red admirals, etc.). Given this taxonomic and functional diversity, it is a reasonable assumption that trends in high‐altitude day‐flying insects are reflective of trends in insect abundance broadly.

### 4.4 Evaluating Insect Trends Requires Long Time Series and an Accurate Baseline

Assessing changes in populations or diversity is inherently conditional on a baseline or the time series starting point, and therefore vulnerable to a false or shifting baseline effect (Pauly 1995; Thomas et al. 2019). Consequently, the net stable insect abundance trend identified in this study may not reflect longer‐term trends if major declines precede our earliest samples. For example, declining trends in terrestrial insect abundance in North America observed by van Klink et al. weakened when evaluating recent decades only (van Klink et al. 2020). In a time series from the US Midwest, a declining trend in aphid abundance was driven by high abundance in the late 2000s (Crossley et al. 2020), and no abundance decline is discerned when analyzing data since 2012 only (Figure S10). Several studies of lepidopterans show that inclusion of high (Macgregor et al. 2019) or low (van Strien et al. 2019) abundances from the 1980s strongly impacts interpretations of more recent declines or increases. Assessing future trends in insect abundance depends both on selecting appropriate timescales for our studies, and the availability of long‐term data to construct a regional historical baseline.

Non‐linear population dynamics characterized by periods of increases and declines are evident in multi‐species insect population studies, regardless of study design, and across a diversity of taxa and geographic regions (Bell et al. 2020; Høye, Loboda, et al. 2021; Macgregor et al. 2019). Insect population dynamics are frequently typified by rapid population surges and declines due to their short generation times and high reproductive capacity (den Boer 1985; Wallner 1987). This propensity for population fluctuations complicates interpretation and necessitates longer time series to evaluate trends. Our 10‐year time series dataset will continue to expand through ongoing automatic weather radar operations, allowing future assessment of whether the net stable trend in insect abundance identified here persists.

### 4.5 The Potential of Radar for Widespread Monitoring

Our study is the most comprehensive approach to date to document the macroscale geography of insect abundance and biomass changes in the United States, and this method has great potential for systematic monitoring of insect abundance across the globe. While existing conventional entomological long time series datasets are mainly located in Europe or North America (van Klink et al. 2020), many countries have existing networks collecting weather radar at widely distributed sites, the data of which are increasingly publicly available. The utility of weather radar has the potential to reach beyond studying abundance to assessing aerial biodiversity changes as well (Shamoun‐Baranes et al. 2021). Lastly, while our methods have only been applied to data since dual‐polarization in 2012, NOAA maintains a multi‐decade historical archive and developing techniques may unlock these data in the near future (Høye, Ärje, et al. 2021; Nussbaumer et al. 2021). Weather surveillance radar data should be integrated with other data streams, such as targeted entomological sampling for species composition, to produce a global insect abundance and diversity monitoring network (Montgomery et al. 2020).

## Author Contributions

**Elske K. Tielens:** conceptualization, data curation, formal analysis, funding acquisition, methodology, visualization, writing – original draft, writing – review and editing. **Phillip M. Stepanian:** data curation, funding acquisition, methodology, writing – review and editing. **Jeffrey F. Kelly:** funding acquisition, supervision, writing – review and editing.

## Conflicts of Interest

The authors declare no conflicts of interest.

## Supporting information


**Data S1:** gcb70587‐sup‐0001‐supinfo.pdf.

## Data Availability

All data used in the study is publicly available. NEXRAD radar data is archived and publicly available from Amazon Web Services: https://noaa‐nexrad‐level2.s3.amazonaws.com. Secondary data and models are available at https://doi.org/10.5281/zenodo.17376559.
