# Supplementary material for: Systematic Continental Scale Monitoring by Weather Surveillance Radar Shows Fewer Insects Above Warming Landscapes in the United States
Source: Glob Chang Biol. 2025 Nov 18;31(11):e70587. doi: 10.1111/gcb.70587 (PMC12625803; doi:10.1111/gcb.70587)
Supplement: Supplementary file 1 — Data S1: gcb70587‐sup‐0001‐supinfo.pdf. [file GCB-31-e70587-s001.pdf]

Supplementary Information for manuscript:
Systematic continental scale monitoring by weather surveillance radar shows fewer insects above warming landscapes in the United States

Authors:
Elske K. Tielens[1]*, Phillip M. Stepanian[2], Jeffrey F. Kelly[1]

[1]Department of Biology, University of Oklahoma, Norman, OK 73019, USA.
[2]Lincoln Laboratory, Massachusetts Institute of Technology, Lexington, MA 02421, USA.

*Corresponding author.
Email: elske.tielens@gmail.com

Author Contributions:
Conceptualization: EKT,
Methodology: PMS, EKT,
Visualization: EKT, PMS,
Funding acquisition: JFK, PMS, EKT
Supervision: JFK
Writing – original draft: EKT
Writing – review & editing: EKT, JFK, PMS

Competing Interest Statement: Authors declare that they have no competing interests.

Keywords: Insect monitoring, global change, urbanization, radar entomology

This PDF file includes:
- Supplementary text
- Figures S1-S12
- Tables S1-S5
- SI References

Supplementary Information Methods

Quality control

As mentioned in the main text, our filtering methods removed the main sources of non-insect signal, and we cleaned the data by calculating weekly mean biomass and removing all data points greater than two standard deviations from the weekly mean. We removed two sites with persistent problems with chaff or other contaminants (KBYX and KAMX). Then, we conducted quality control and assessed the accuracy of our filtering by (1) evaluating outliers; and (2) testing predictions of expected insect abundance.

To evaluate outliers we identified >700 high values and visually inspected raw scans to assess the source of scatter, removing data points when filtered scan showed non-insect scatter. We evaluated shape or pattern of the scatter, compared across radar products (i.e. diff reflectivity, correlation coefficient), examined the direction and speed of movement, and its location on the landscape relative to possible sources of false positives. We were conservative in our estimates and excluded scans if there was uncertainty about the accuracy of the datapoint.

As the next step in our quality check process, we conducted a series of 'internal checks' to verify that our data meets general predictions for insect abundance at macroecological scales. We evaluated the latitudinal gradient in insect abundance, winter insect abundance in temperate regions, and the relationship between abundance and temperature.

Across latitudes there is a gradient in solar energy input, net primary productivity, and the length of the growing season. These factors can be expected to result in a latitudinal gradient in insect abundance. We confirmed this relationship by regressing mean annual insect density on latitude, showing a strong negative relationship ($F_{1,138}$ = 31.7, adj. R^2 = 0.18, p < 0.0001).

Physiologically constrained activities, including insect flight, are a function of temperature, such that warmer sites should have greater insect flight activity (within thermal maxima). We tested this prediction by regressing annual mean site density on air temperature and found that 46% of year to year and site to site variation in summed density was explained by mean summer temperature (linear model of log insect density as a function of mean summer daily temperature, adj. R^2=0.46, p<0.001). Similarly, in our gradient boosted regression tree model surface air temperature was one of the top features in explaining day-to-day and site-to-site variation in observed insect abundance (Figure S2).

At higher latitudes insect activity nearly ceases during the winter, resulting in the prediction that winter aerial insect density should approach zero in temperature regions. In our dataset, mean daily insect density during the winter for sites at latitudes above 40° was 1.99 insects m^{-2}. This is visible also in site-specific patterns of seasonality in our data, where temperate sites show a strong seasonality in insect density, and lower latitude sites show a less unimodal distribution (Fig. S4A,B). Mean daily winter insect density decreased with latitude ($F_{1,137}$ = 42.36, adj. R^2=0.24, p<0.00001).

Data analysis

To assess drivers of insect density anomaly and insect density trends we used two different response variables; annual insect density anomaly and 10-year trend in insect density. We used a model averaging approach based on linear regressions to assess climate and land cover drivers. Model selection and averaging was done using the package 'MuMIn' in R.

We assessed the role of climate in driving interannual variability by model selection of a mixed effect model with 30-year temperature anomaly for spring, summer, fall, and winter as well as precipitation anomaly, including latitude, longitude, and a random effect for site ID.

We assessed trends in insect density for each site for the 10-year period by taking the coefficient of site specific linear regression models across the entire time period. The advantage of this approach is that it smooths out high interannual variation typical of insect density data while maintaining the intrinsic data structure. To take into account site to site variation in overall insect density (i.e. to prevent high

declines at sites with low overall insect activity from skewing the results), we scale by annual summed density to convert the slope coefficient to percentage change. Percent change data is frequently characterized by positive skew, which may violate error assumptions in linear regression models (i.e. with Gaussian error distribution). We tested for positive skew and found that our data indeed has a longer right tail (skewness=1.17). This skew is the result of two outliers (KBLX & KGGW; excluding these values skewness=0.057). We further assessed assumptions underlying linear regression models including normal distribution of residuals and heteroscedasticity. Similar to the results on skewness, we found that these two outliers affected the residuals, and that after removing them the assumptions for linear regression were met (i.e. for full climate model, shapiro wilkinson test W = 0.98705, p-value = 0.2264). While log transformation could be used to address skew in percentage change data, we preferred to conduct analyses with minimal data transformation as possible to avoid distorting the original relationships between variables and maintain an additive rather than multiplicative relationship. To assess whether the effect of these outliers on model fit and assumptions would change model interpretation, we re-analyzed the data with the outliers for KBLX and KGGW omitted. We found qualitatively similar results; thus we concluded that our results were robust to any statistical issue introduced by these outliers. We then proceeded with the original dataset including KBLX and KGGW, without data transformation. To assess climate and land cover drivers, we generated model selection tables and then conducted model averaging for coefficient estimation based on AIC (averaging all models within 4 AIC). We generated full models for climate and land use separately to prevent multicollinearity. In addition to the predictors described in the main article, we analyzed the role of human footprint index, population density, biome, net primary productivity, and several weather variables.

To further explore potential anthropogenic causes of insect abundance trends we analyzed two other predictors for human development; the Human Footprint index and population density (Table S1, Fig. S6). The Human Footprint Index is a metric of cumulative human pressure on the environment (Venter et al., 2018), based on eight variables including population density, built-up environments, land use and roads. This data was compiled in 2009 at a spatial resolution of ~1 km. We quantified HFP at a scale matching the radar insect density data by calculating the mean for an 80 km radius around the radar site. To quantify population density, we used 2020 data from the NASA SEDAC Gridded population of the world at 2.5 arc-minute scale (SEDAC, 2018), and calculated mean population density within an 80 km radius of the site.

To evaluate the role of vegetation as a predictor of change in insect abundance we explored both biome and net primary productivity (Table S1, Fig. S7 and S8). We used data from the WWF Terrestrial Ecoregions of the World to identify biomes for each site (Olson et al., 2001). Annual net primary productivity was given by NASA MODIS satellite at 500 meter pixel resolution, and is derived from the sum of all 8-day GPP Net Photosynthesis products for a given year (Running, S. & Zhao, M., 2021). We calculated median productivity across all cells within a 50 km^2 grid centered on the radar station.

We extracted monthly data on local climatic conditions from WorldClim for each radar station for the period 2012-2021 (Fick & Hijmans, 2017). We used the slope of site-specific linear regressions to calculate the 10-year change in mean, minimum, and maximum spring temperature, summer temperature, fall temperature, and winter temperature, as well as the 10-year change in mean annual precipitation. We analyzed the role of change in weather variables for change in insect density using linear regression. We explored the role of mean, minimum, and maximum temperature for each season. We decided to use the temporal trend in mean temperature after comparing results from regressing insect density trends against mean, minimum, and maximum temperature trends and finding comparable results (Table S2).

Suction trap network

To explore the role of baselines in analyzing long term abundance of insects, we explored aphid abundance collected using the soybean aphid Suction Trap Network (STN), a publicly available dataset

(Crossley et al., 2020) that contains data on several species of aphids collected in the United States Midwest during the period 2006-2019. More information on suction traps, the network, and how samples are collected can be found here (Lagos-Kutz et al., 2020). We calculated annual summed totals per suction trap (site), and conducted linear regression on these annual sums. We analyzed temporal trends for the complete time period, and then we analyzed temporal trends for the time period for which we have radar data available (i.e. from 2012 onwards). Fitted lines were derived from least-square linear regression on year, based on the complete time period (Fig. S11, dashed line, Est.= -162.4, $p < 0.0001$) and for the period 2012-2019 only (Fig. S11, solid line, Est. = 15.8, $p = 0.42$).

To compare our results to another multi-site analysis of aerial insects, we analyzed temporal trends in the suction trap network's data and in a regional subset of our radar data. This cannot serve as a true comparison due to differences in scope and sampling frequency, among other reasons: suction traps collect cumulative totals on a weekly basis, where we extract daily snapshots of insect density from the radar archive; suction traps sample the air only at 10 m above the ground, which is below the altitude where radar observations are available; and the STN predominantly collects aphids (Hemiptera) while radar observations include any day-flying insects across a wide range of taxa and orders. However, the STN is a regional dataset of aerial insects that provides the closest comparison to radar observations, and we show here that temporal trends from the STN dataset are broadly similar to conclusions based on radar data. To make this comparison, we restricted radar observations to the Midwest region based on coordinates of the STN sites (latitude between 35.43 and 48.77, longitude between -83.83 and -97.67), and we restricted data to the years for which both data types were available (2012-2019). We quantified annual insect density on radar per site by summing across daily noontime scans, and for STN samples we summed total abundance collected per site. We centered and scaled these values independently for the two data types to create z-scores, using the function 'scale' in R. We analyzed the temporal trend for both data types separately using least-squares linear regression. Both analyses found no significant temporal trend in insect density (Fig. S12, radar Est.= 0.35, $p = 0.32$; STN Est. = -0.0073, $p = 0.42$).

SI References

Center for International Earth Science Information Network. (2018). *Gridded Population of the World, Version 4 (GPWv4): Population Count, Revision 11*. NASA Socioeconomic Data and Applications Center (SEDAC). https://doi.org/10.7927/H4JW8BX5

Crossley, M. S., Meier, A. R., Baldwin, E. M., Berry, L. L., Crenshaw, L. C., Hartman, G. L., Lagos-Kutz, D., Nichols, D. H., Patel, K., Varriano, S., Snyder, W. E., & Moran, M. D. (2020). No net insect abundance and diversity declines across US Long Term Ecological Research sites. *Nature Ecology & Evolution*, *4*(10), 1368–1376. https://doi.org/10.1038/s41559-020-1269-4

Dewitz, J. (2019). *National Land Cover Database (NLCD) 2016 Products*. U.S. Geological Survey data release. https://doi.org/10.5066/P96HHBIE

Fick, S. E., & Hijmans, R. J. (2017). WorldClim 2: New 1-km Spatial Resolution Climate Surfaces for Global Land Areas. *International Journal of Climatology*, *37*, 4302-4315.

Lagos-Kutz, D., Voegtlin, D. J., Onstad, D., Hogg, D., Ragsdale, D., Tilmon, K., Hodgson, E., Difonzo, C., Groves, R., Krupke, C., Laforest, J., Seiter, N. J., Duerr, E., Bradford, B., & Hartman, G. L.

(2020). The Soybean Aphid Suction Trap Network: Sampling the Aerobiological "Soup." *American Entomologist*, *66*(1), 48–55. https://doi.org/10.1093/ae/tmaa009

Olson, D. M., Dinerstein, E., Wikramanayake, E. D., Burgess, N. D., Powell, G. V. N., Underwood, E. C., D'amico, J. A., Itoua, I., Strand, H. E., Morrison, J. C., Loucks, C. J., Allnutt, T. F., Ricketts, T. H., Kura, Y., Lamoreux, J. F., Wettengel, W. W., Hedao, P., & Kassem, K. R. (2001). Terrestrial Ecoregions of the World: A New Map of Life on Earth. *BioScience*, *51*(11), 933–938. https://doi.org/10.1641/0006-3568(2001)051[0933:TEOTWA]2.0.CO;2

Running, S. & Zhao, M. (2021). *MODIS/Terra Net Primary Production Gap-Filled Yearly L4 Global 500m SIN Grid V061*. NASA EOSDIS Land Processes DAAC. https://doi.org/10.5067/MODIS/MOD17A3HGF.061

Venter, O., Sanderson, E. W., Magrach, A., Allan, J. R., Beher, J., Jones, K. R., Possingham, H. P., Laurance, W. F., Wood, P., Fekete, B. M., Levy, M. A., & Watson, J. E. (2018). *Last of the Wild Project, Version 3 (LWP-3): 2009 Human Footprint, 2018 Release*. NASA Socioeconomic Data and Applications Center (SEDAC). https://doi.org/10.7927/H46T0JQ4

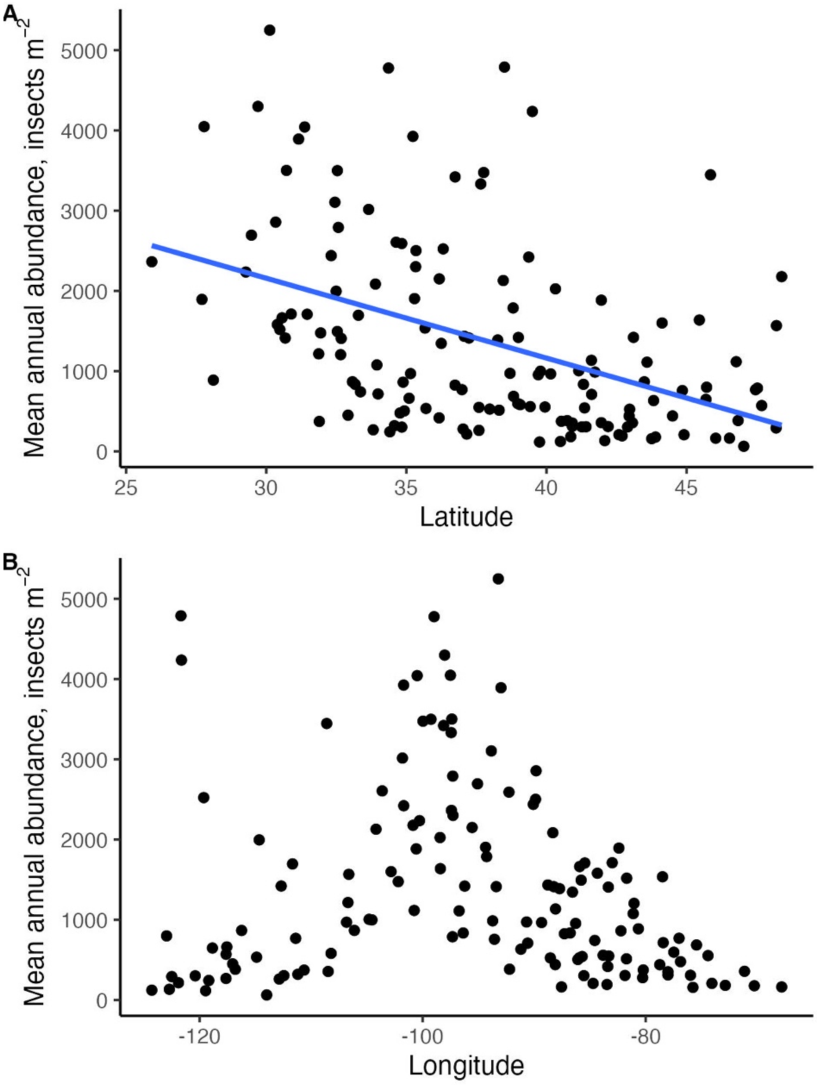

Fig. S1. Mean annual insect density per site in insects m^{-2} as a function of latitude (A), and longitude (B). The line is given by least-square regression, est.=-3.14, $p<0.0001$, adj. R^2=0.19.

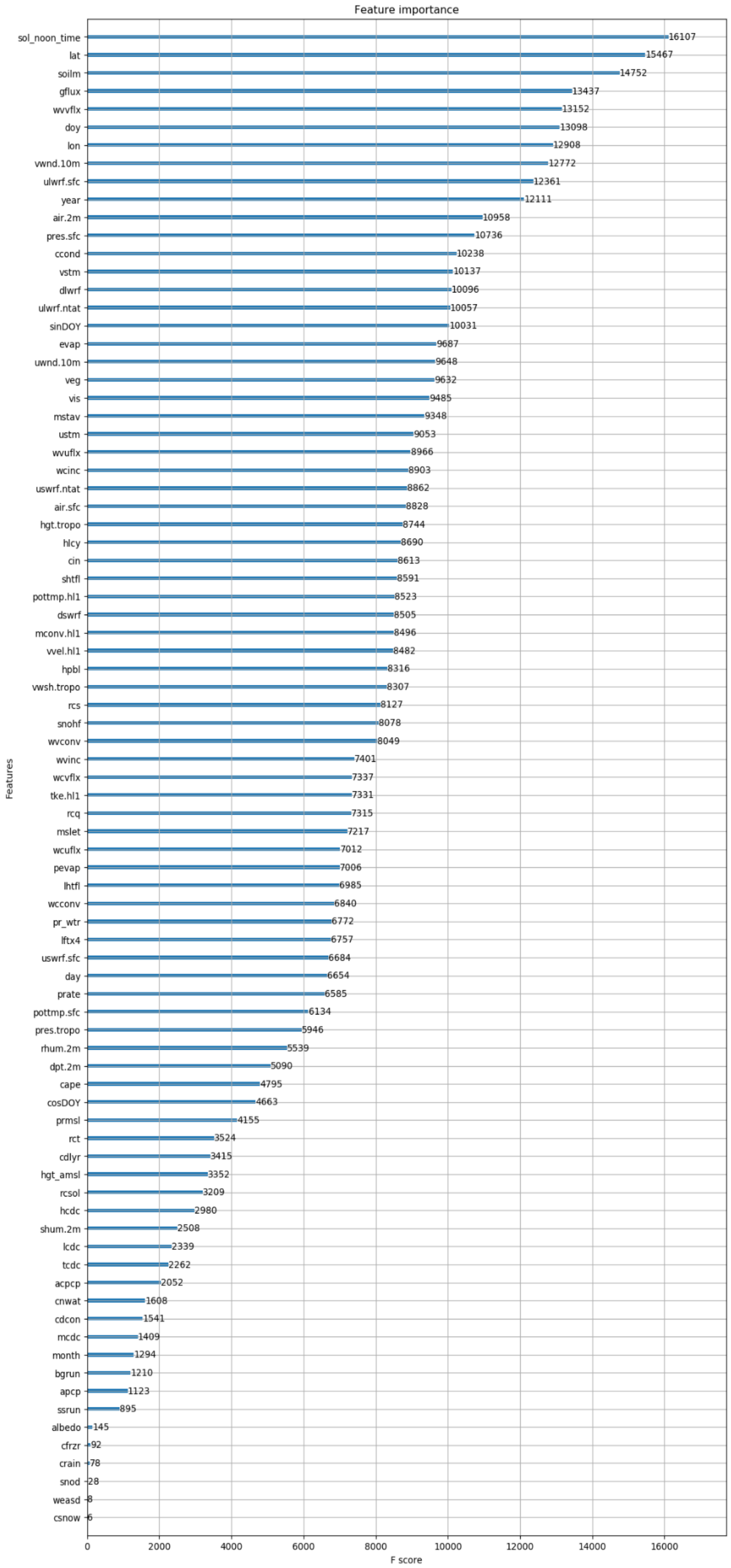

Fig. S2. Feature importance for the gradient-boosted regression tree model. F-scores for all NARR predictor variables included in full model. Explanation of NARR variables is found at (56).

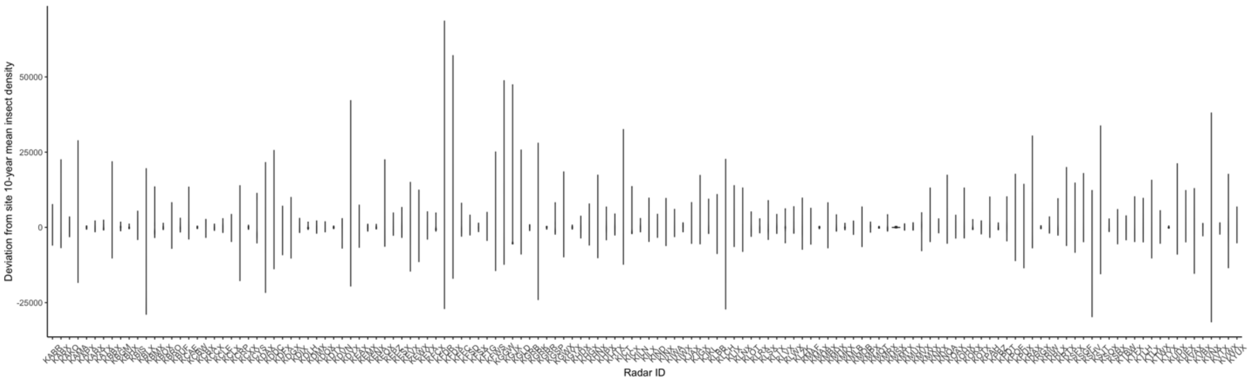

Fig. S3. Deviation from 10-year mean insect annual density (insects m^{-2}) per site.

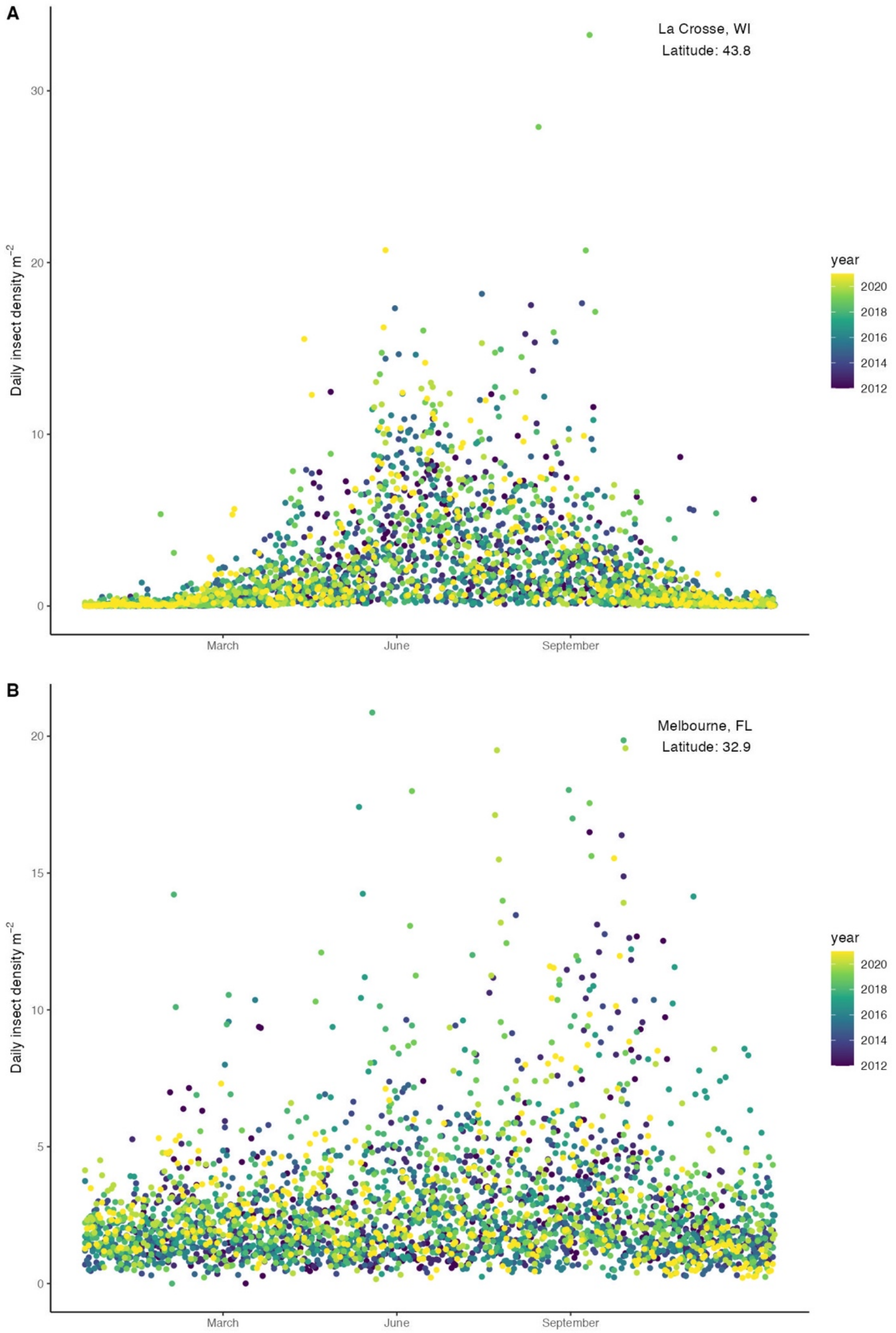

Fig. S4. Seasonal pattern in daily insect density in insects m^{-2} at temperate latitude (A), and lower latitude (B). Color indicates sampling year. The temperate site is La Crosse, Wisconsin (KARX, latitude: 43.8, longitude: -91.2). The lower latitude site is Melbourne, Florida (KMLB, lat: 28.1, lon: -80.7).

Change in day-flying insect density over 2012-2021

Table S1. Model output of landscape associations of trend in insect density. Model estimates from linear regression of percentage change in 10-year day-flying insect density on individual land cover types and environmental predictors. Each predictor was modeled separately in a linear regression with only latitude, longitude, and the environmental predictor. Significant predictors are bolded and marked by * (<0.05), ** (<0.01), and non-significant trends (0.1 < p > 0.05) are marked by °.

	Land cover fraction developed	Land cover fraction crop	Land cover fraction pasture	Land cover fraction forest	Land cover fraction grassland	Land cover fraction shrub	Human footprint index	Population density	Biome	Net primary productivity
**Model estimate**	**-15.2***	-1.99	-4.36	5.40°	6.22°	-1.69	**-0.24***	-0.0002	NS	-0.00016

Table S2. Model output of climatic associations of trend in insect density. Model estimates from linear regression of percentage change in 10-year day-flying insect density on the change in 10-year mean, minimum, and maximum spring, summer, fall, and winter temperatures, as well as mean annual precipitation. Significant predictors are bolded and marked by *, and non-significant trends (0.1 < p > 0.05) are marked by °. Outliers were omitted in calculating model coefficients. Omitting outliers did not qualitatively change results or effect size direction, and only weakened effect sizes and existing significance levels.

	Change in winter temperature	Change in spring temperature	Change in summer temperature	Change in fall temperature	Change in annual precipitation
**Mean**	**-22.09***	-8.86	-2.26	-11.79	-4.80
**Minimum**	**-11.71***	-6.67	-8.25	-8.50	NA
**Maximum**	**-8.00***	-5.95	2.56	-7.80	NA

Table S3. Correlation coefficients between predictors. Correlation coefficients between change in mean winter temperature and fraction land cover for most common NLCD land cover types.

	Correlation coefficient	p-value
Fraction developed cover	0.353	$1.88*10^{-5}$
Fraction cropland cover	-0.112	0.187
Fraction pasture cover	0.0897	0.292
Fraction forest cover	0.419	$2.5*10^{-7}$
Fraction grassland cover	-0.490	$7.67*10^{-10}$
Fraction shrubland cover	-0.307	0.000221
Fraction aquatic cover	0.253	0.00256

Table S4: Pearson partial correlation coefficient between the trend in insect density and the trend in mean winter temperature while controlling for land cover variables (left), and partial correlation coefficient between the trend in insect density and fraction land cover while controlling for the trend in mean winter temperature (right). P-values are given * (<0.05), ** (<0.01), *** (<0.001).

	Partial correlation with winter temperature	Partial correlation with fraction land cover
Fraction developed	-0.244**	-0.185*
Fraction grassland	-0.236**	0.100

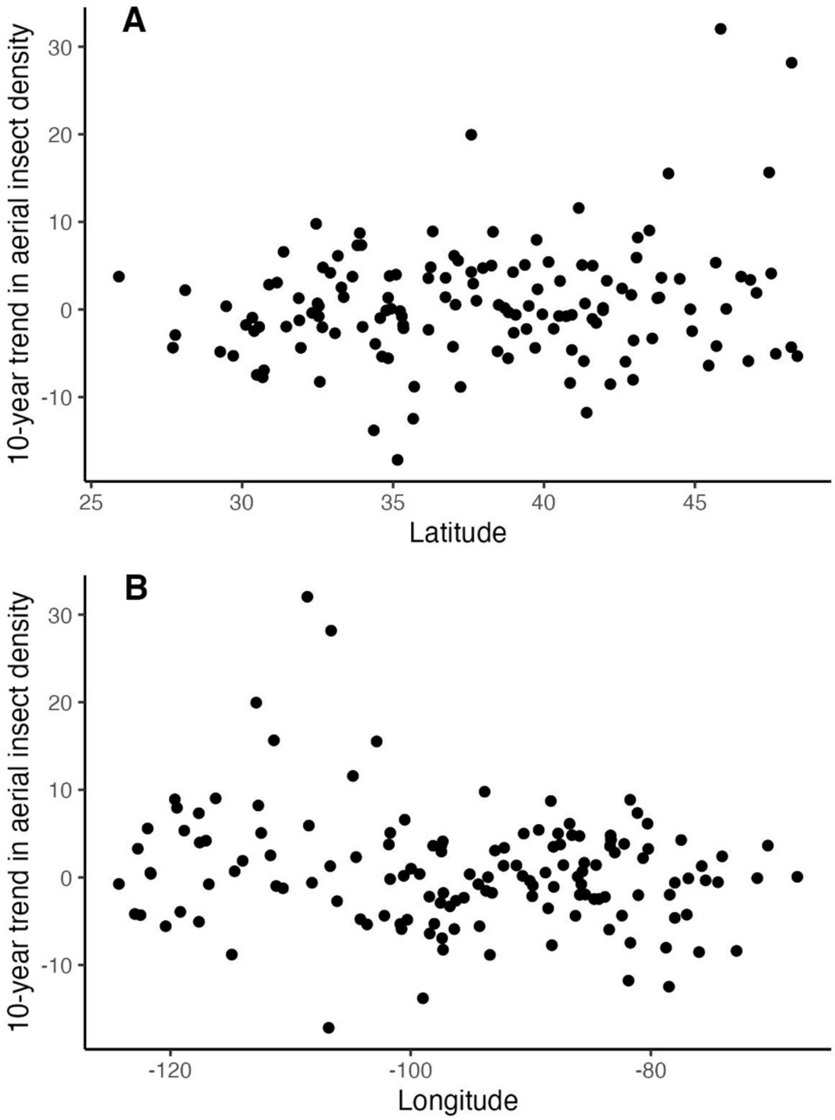

Fig. S5. Percentage change in insect density as a function of latitude (A) and longitude (B). 10-year percentage change in day-flying insect density per latitude and longitude (latitude: estimate = 0.23, p = 0.077, longitude: estimate = -0.071, p = 0.14).

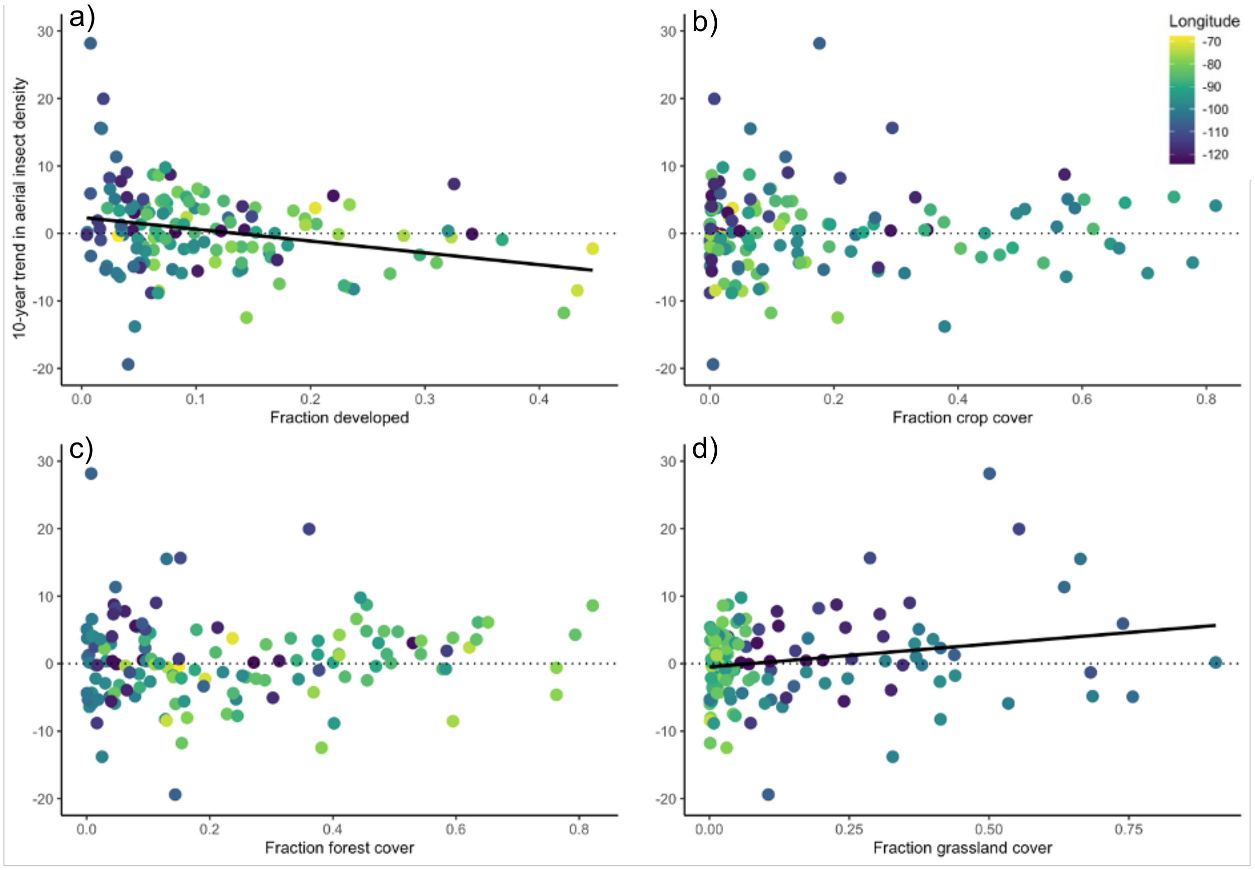

Figure S6. Change in insect density as a function of land cover. 10-year trend in day-flying insect density as a function of the most common land cover types, colored by site longitude. Temporal trend in insect density is shown along fraction developed land cover (a), fraction crop cover (b), fraction forest cover (c) and fraction grassland (d). Land cover type is based on 2016 NLCD land cover (Dewitz, 2019), with fraction of land cover calculated within an 80 km radius to the radar. Fitted line is derived from a least-square linear regression on percentage change in insect density (developed area fraction est.=-19.8, $p<0.001$; grassland fraction est.=8.1, $p<0.01$).

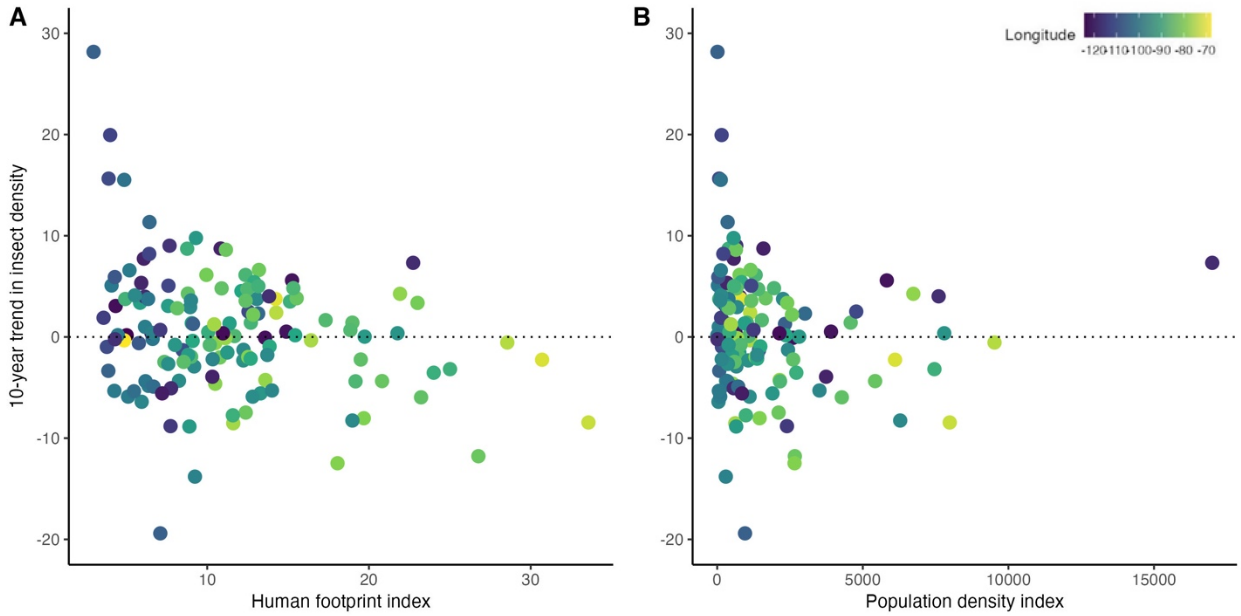

Fig. S7. Change in insect density as a function of human footprint index and population density. 10-year trend in day-flying insect density as a function of (A) human footprint index (19) and (B) population density index (50), colored by site longitude. Fitted line is derived from a least-square linear regression on percentage change in insect density.

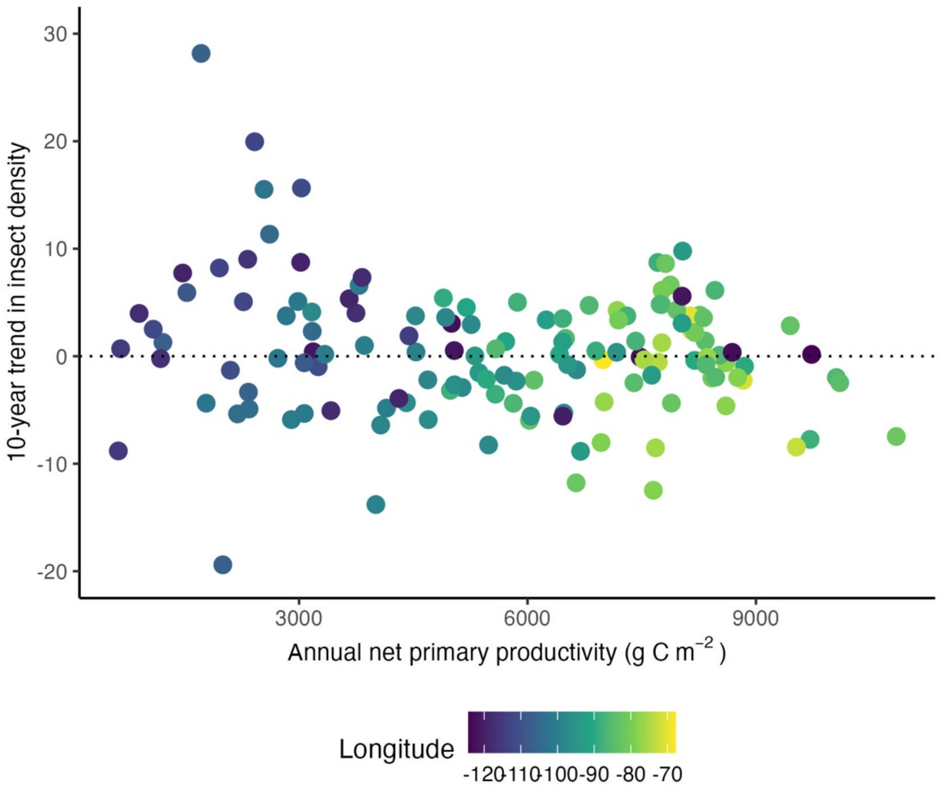

Fig. S8. Change in insect density as a function of net primary productivity. 10-year trend in day-flying insect density as a function of annual net primary productivity (52), colored by site longitude.

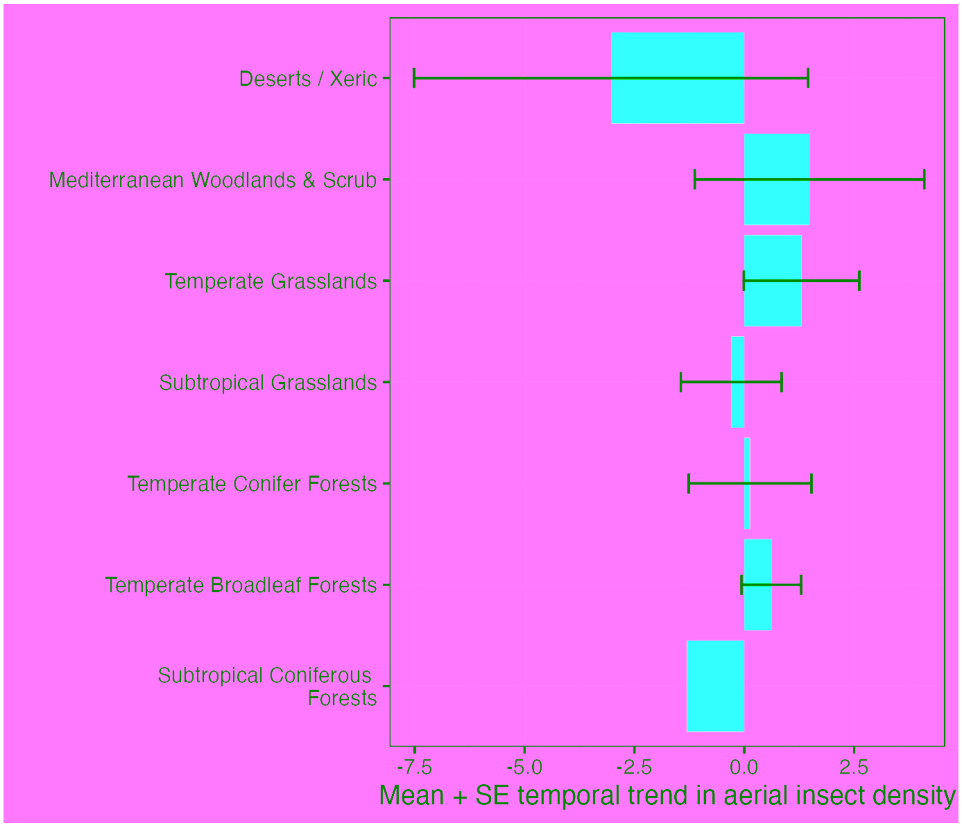

Fig. S9. Mean and standard error of trend in insect density as a function of site biome. 10-year trend in day-flying insect density across biomes at the radar site. Standard error is not given for biomes occurring at a single site.

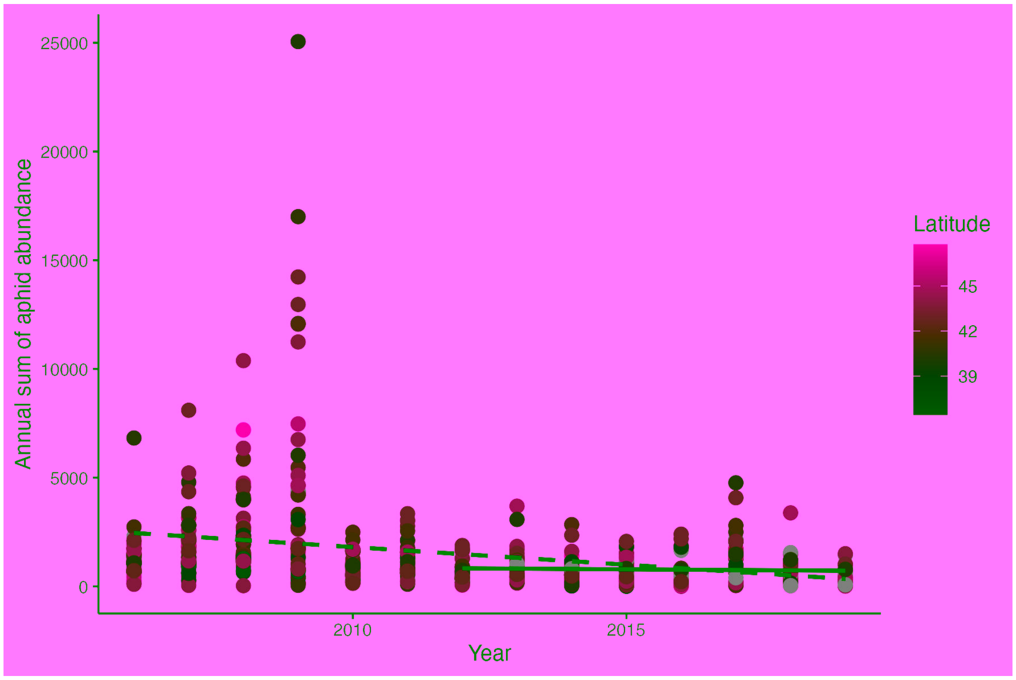

Fig. S10. Insect abundance in suction trap data. Summed annual aphid abundance per suction trap over the period 2006-2019, derived from publicly available data (8) collected by the Suction Trap Network (55). Dots are colored by suction trap site latitude. Fitted lines are derived from least-square linear regression on year, based on the complete time period (dashed line, Est.= -162.4, $p < 0.0001$) and for the period 2012-2019 only (solid, Est. = 15.8, $p = 0.42$).

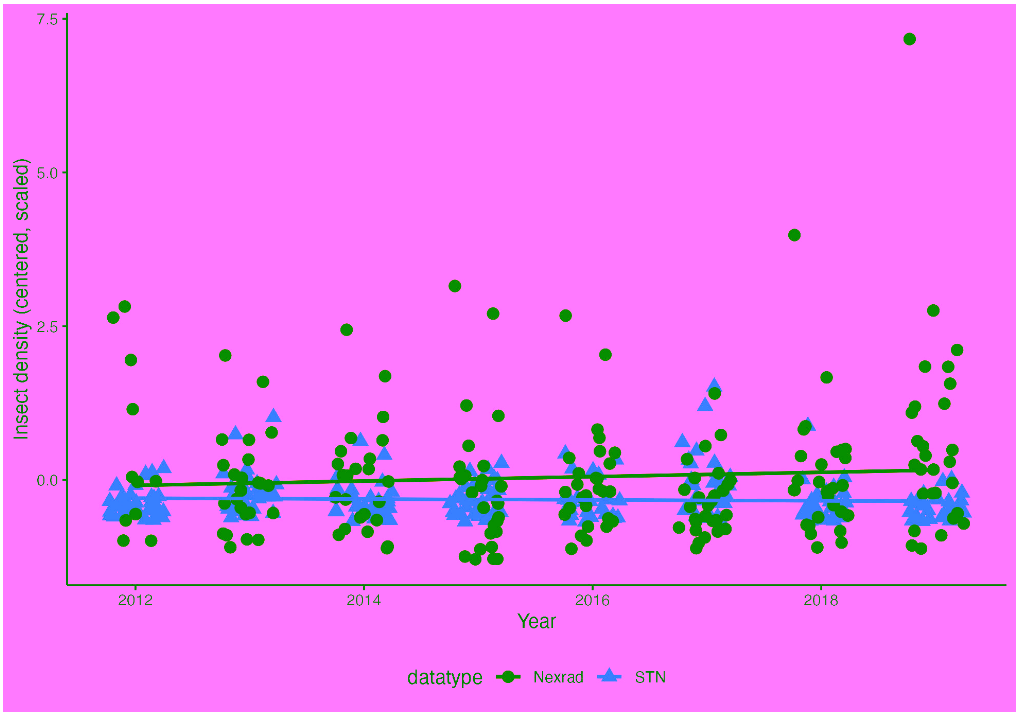

Fig. S11. Scaled and centered insect abundance in radar and suction trap data over time. Comparison of centered and scaled insect abundance between NEXRAD radar observations (purple circles) and Suction Trap Network sampling (8) (green triangles) in the Midwest region for the period 2012-2019. Insect density on radar was summed annually per site across daily noontime scans, and STN aphid abundance was summed for each suction trap. Both data types were independently centered and scaled to create z-scores. Fitted lines were derived from least-square linear regression on year (NEXRAD radar in purple, Est.= 0.35, p = 0.32; STN in green, Est. = -0.0073, p = 0.42). Points were jittered on the x-axis for visibility.

Table S5. Site information. Radar site code, latitude, longitude, date of dual polarization and percentage change in insect density in the 10-year study period.

ID	Latitude	Longitude	Date of activation dual polarization	10-year percentage change
KABR	45.45583	-98.41306	7-Aug-12	-6.40
KABX	35.14972	-106.82389	7-May-12	-19.40
KAKQ	36.98389	-77.00722	15-Feb-12	-4.25
KAMA	35.23333	-101.70917	14-Nov-11	-0.19
KAPX	44.90722	-84.71972	28-Nov-11	-2.47
KARX	43.82278	-91.19111	18-Apr-12	1.36
KATX	48.19444	-122.49583	6-Oct-11	-0.08
KBBX	39.49611	-121.63167	16-Mar-12	0.41
KBGM	42.19972	-75.98472	9-Apr-12	-8.53
KBHX	40.49833	-124.29194	24-May-12	0.17
KBIS	46.77083	-100.76056	27-Jul-12	-5.89
KBLX	45.85389	-108.60667	8-Nov-11	32.04
KBMX	33.17222	-86.76972	13-Feb-12	6.12
KBOX	41.95583	-71.13694	11-Jan-12	-2.25
KBRO	25.91611	-97.41889	21-Apr-13	3.75

KBUF	42.94889	-78.73667	2-Apr-12	-8.03
KCAE	33.94861	-81.11833	7-May-12	6.62
KCBW	46.03917	-67.80639	7-Aug-12	-0.34
KCBX	43.49056	-116.23556	13-Sep-12	9.02
KCCX	40.92306	-78.00361	23-Mar-12	-4.62
KCLE	41.41306	-81.85972	8-Dec-11	-11.78
KCLX	32.65556	-81.04222	25-Oct-12	-2.03
KCRP	27.78417	-97.51111	8-Apr-13	-2.91
KCXX	40.92306	-78.00361	27-Jul-12	-0.61
KCYS	41.15194	-104.80611	10-Oct-12	11.35
KDAX	38.50111	-121.67778	27-Jun-12	0.52
KDDC	37.76083	-99.96889	18-Jan-12	1.00
KDFX	29.27278	-100.28056	29-Oct-12	-4.83
KDGX	32.31778	-90.08	23-Jan-13	-0.39
KDIX	39.94694	-74.41083	31-Jan-12	-0.55
KDLH	46.83694	-92.20972	14-May-12	3.38
KDMX	41.73111	-93.72278	14-Sep-12	-1.53
KDOX	38.82556	-75.43972	8-Feb-12	-0.33

KDTX	42.69972	-83.47167	27-Mar-13	-5.96
KDVN	41.61167	-90.58083	26-Mar-12	4.53
KDYX	32.53833	-99.25444	10-Apr-13	0.40
KEAX	38.81028	-94.26444	10-Feb-12	-5.57
KEMX	31.89361	-110.63028	18-Jan-12	-1.31
KENX	42.58639	-74.06389	30-Apr-12	2.40
KEOX	31.46056	-85.45944	9-Feb-13	-1.95
KEPZ	31.87306	-106.69806	11-Jun-12	1.29
KESX	35.70111	-114.89139	31-Mar-12	-8.81
KEVX	30.56444	-85.92139	13-Jan-13	-1.99
KEWX	29.70389	-98.02833	3-Apr-12	-5.29
KEYX	35.09778	-117.56083	22-Feb-12	3.98
KFCX	37.02444	-80.27389	11-Oct-12	6.13
KFDR	34.36222	-98.97639	15-Oct-12	-13.80
KFDX	34.63528	-103.63	26-Apr-12	-5.37
KFFC	33.36361	-84.56583	13-Dec-11	1.41
KFSD	43.58778	-96.72944	30-Jul-12	-4.33
KFSX	34.57444	-111.19778	2-Oct-12	-0.98

KFTG	39.78667	-104.54583	13-Sep-12	2.31
KFWS	32.57306	-97.30306	16-Nov-12	-8.26
KGGW	48.20639	-106.625	20-Aug-12	28.17
KGJX	39.06222	-108.21389	30-Apr-12	-0.61
KGLD	39.36694	-101.70028	8-Dec-11	5.09
KGRB	44.49833	-88.11139	30-Apr-12	3.50
KGRK	30.72194	-97.38306	7-Nov-12	-1.27
KGRR	42.89389	-85.54472	8-Dec-11	1.66
KGSP	34.88333	-82.22	18-Oct-12	3.82
KGWX	33.89667	-88.32889	5-Nov-12	8.72
KGYX	43.89139	-70.25639	1-Aug-12	3.76
KHDX	33.07639	-106.12278	29-May-12	-3.35
KHGX	29.47194	-95.07917	21-Jan-13	0.37
KHNX	36.31417	-119.63222	30-Jul-12	8.74
KHPX	36.73667	-87.285	1-Feb-13	1.41
KHTX	34.93056	-86.08333	11-Jan-12	0.09
KICT	37.65472	-97.44278	19-Jul-11	2.95
KICX	37.59083	-112.86222	20-Jun-12	19.95

KILN	39.42028	-83.82167	30-Aug-12	-2.23
KILX	40.15056	-89.33694	10-Oct-12	5.41
KIND	39.7075	-86.28028	19-Oct-12	-4.38
KINX	36.175	-95.56472	19-Sep-12	-2.32
KIWA	33.28917	-111.67	7-Jun-11	2.51
KIWX	41.35889	-85.7	15-Feb-13	0.68
KJAX	30.48472	-81.70194	24-Feb-12	-7.47
KJGX	32.67528	-83.35111	24-Feb-12	4.80
KJKL	37.59083	-83.31306	8-Nov-12	4.28
KLBB	33.65417	-101.81417	27-Mar-13	3.75
KLCH	30.12528	-93.21583	21-Feb-13	-1.77
KLIX	30.33667	-89.82556	27-Mar-13	-0.93
KLNX	41.95778	-100.57639	18-Apr-13	0.18
KLOT	41.60472	-88.08472	31-Oct-11	-3.18
KLRX	40.73972	-116.80278	20-Aug-12	-0.22
KLSX	38.69889	-90.68278	27-Feb-12	0.17
KLTX	33.98944	-78.42889	7-Jun-12	-1.98
KLVX	37.97528	-85.94389	25-Oct-12	4.73

KLWX	38.97528	-77.47778	27-Feb-12	4.26
KLZK	34.83639	-92.26222	27-Aug-12	1.34
KMAF	31.94333	-102.18917	4-Apr-13	-4.37
KMAX	42.08111	-122.71722	20-Aug-12	3.06
KMBX	48.3925	-100.865	20-Jul-12	-5.33
KMHX	34.77611	-76.87611	28-Jun-11	-0.12
KMKX	42.96778	-88.55056	3-Apr-12	-3.53
KMLB	28.11333	-80.65417	27-Jan-12	2.20
KMOB	30.67944	-88.23972	22-Jan-13	-7.74
KMPX	44.84889	-93.56556	15-Aug-12	0.03
KMQT	46.53111	-87.54833	7-May-12	3.75
KMRX	36.16861	-83.40167	30-Jan-12	3.58
KMSX	47.04111	-113.98611	31-Jul-12	1.90
KMTX	41.26278	-112.44778	10-Oct-12	5.07
KMUX	37.15528	-121.89833	26-Apr-12	5.59
KMVX	47.52778	-97.32556	24-May-12	4.11
KMXX	32.53667	-85.78972	18-Feb-13	-0.79
KNKX	32.91889	-117.04194	20-Jul-12	4.02

KNQA	35.34472	-89.87333	13-Dec-11	-2.14
KOAX	41.32028	-96.36667	28-Mar-13	-5.90
KOHX	36.24722	-86.5625	27-Jan-12	4.82
KOKX	40.86556	-72.86389	20-Jan-12	-8.45
KOTX	47.68028	-117.62667	25-Oct-11	-5.07
KPAH	37.06833	-88.77194	13-Jan-13	0.49
KPBZ	40.53167	-80.21806	19-Jul-11	3.37
KPDT	45.69056	-118.85278	6-Oct-11	5.34
KPOE	31.15556	-92.97583	8-Feb-13	3.07
KPUX	38.45944	-104.18139	3-Apr-13	-4.91
KRAX	35.66556	-78.48972	13-Nov-12	-12.47
KRGX	39.75417	-119.46222	4-Sep-12	7.74
KRIW	43.06611	-108.47722	28-Nov-11	5.92
KRLX	38.31111	-81.72306	2-Oct-12	8.61
KRTX	45.71472	-122.96528	27-Sep-11	-4.18
KSFX	43.10583	-112.68611	20-Oct-12	8.21
KSGF	37.23528	-93.40056	17-Feb-12	-8.85
KSHV	32.45083	-93.84139	1-Feb-13	9.78

KSJT	31.37139	-100.4925	19-Apr-12	6.58
KSOX	33.81778	-117.63583	25-Jan-12	7.33
KSRX	35.29056	-94.36167	11-Sep-12	-0.78
KTBW	27.70556	-82.40167	8-Mar-12	-4.36
KTFX	47.45972	-111.38528	23-Jul-12	15.65
KTLH	30.3975	-84.32889	25-Oct-12	-2.46
KTLX	35.33306	-97.27778	8-Oct-12	-1.78
KTWX	38.99694	-96.2325	30-Jan-12	-2.66
KTYX	43.75583	-75.76333	20-Jul-12	1.25
KUDX	44.125	-102.82972	19-Oct-12	15.52
KUEX	40.32083	-98.44194	6-Apr-13	-2.20
KVAX	30.89028	-83.00167	16-May-13	2.84
KVBX	34.83806	-120.39694	6-Feb-12	-5.57
KVNX	36.74083	-98.12778	8-Mar-11	3.61
KVTX	34.41167	-119.17944	10-Apr-12	-3.93
KYUX	32.49528	-114.65667	10-May-12	0.70
KVWX	38.26039	-87.72465	9-Feb-13	5.02
